# TBESO-BP: an improved regression model for predicting subclinical mastitis

**DOI:** 10.3389/fvets.2025.1396799

**Published:** 2025-04-01

**Authors:** Kexin Han, Yongqiang Dai, Huan Liu, Junjie Hu, Leilei Liu, Zhihui Wang, Liping Wei

**Affiliations:** ^1^College of Information Science and Technology, Gansu Agricultural University, Lanzhou, China; ^2^College of Veterinary Medicine, Gansu Agricultural University, Lanzhou, China; ^3^Gansu Nongken Tianmu Dairy Co., Ltd., Jinchang, China

**Keywords:** subclinical mastitis, snake optimization, regression prediction, neural network, Dairy Herd Improvement

## Abstract

**Introduction:**

Subclinical mastitis in dairy cows carries substantial economic, animal welfare, and biosecurity implications. The identification of subclinical forms of the disease is routinely performed through the measurement of somatic cell count (SCC) and microbiological tests. However, their accurate identification can be challenging, thereby limiting the opportunities for early interventions. In this study, an enhanced neural backpropagation (BP) network model for predicting somatic cell count is introduced. The model is based on TBESO (Multi-strategy Boosted Snake Optimizer) and utilizes monthly Dairy Herd Improvement (DHI) data to forecast the status of subclinical mastitis in cows.

**Materials and methods:**

The Monthly Dairy Herd Improvement (DHI) data spanning from January 2022 to July 2022 (full dataset) was partitioned into both the training and testing datasets. TBESO addresses the challenge associated with erratic initial weights and thresholds in the BP neural network, impacting training outcomes. The algorithm employs three strategies to rectify issues related to insufficient population diversity, susceptibility to local optimization, and reduced accuracy in snake optimization. Additionally, six alternative regression prediction models for subclinical mastitis in dairy cows are developed within this study. The primary objective is to discern models by exhibiting higher predictive accuracy and lower error values.

**Results:**

The evaluation of the TBESO-BP model in the test phase reveals a coefficient of determination *R*^2^ = 0.94, a Mean Absolute Error (MAE) of 2.07, and a Root Mean Square Error (RMSE) of 5.33. In comparison to six alternative models, the TBESO-BP model demonstrates superior accuracy and lower error values.

**Discussion:**

The TBESO-BP model emerges as a precise tool for predicting subclinical mastitis in dairy cows. The TBESO algorithm notably enhances the efficacy of the BP neural network in regression prediction, ensuring elevated computational efficiency and practicality post-improvement.

## 1 Introduction

Mastitis stands out as the most costly and prevalent ailment affecting dairy cows, leading to a substantial decline in milk yield and impacting lactation function. The consequence often involves the culling of affected cows, resulting in a significant financial setback for dairy farms (1). Research indicates that mastitis in dairy cows accounts for approximately 38% of the total direct expenditure associated with common production diseases on dairy farms (2). Notably, subclinical mastitis, being 40 times more prevalent than clinical mastitis and challenging to detect, accentuates its pronounced economic impact (3). Consequently, the imperative lies in the development and utilization of efficient models for the prediction and diagnosis of subclinical mastitis. Presently, the primary methods employed for detecting subclinical mastitis on farms include somatic cell count (SCC) (4), the California mastitis test (5), milk pH testing (6), milk conductivity testing (7), milk enzyme analysis (5) and pathogen diagnosis (8). Despite offering reasonable accuracy in indicating subclinical mastitis, these methods are often associated with a substantial workload and extended intervals between tests. Due to their inherent limitations, these methods are unlikely to meet the farm’s demand for accurate prediction of subclinical mastitis (9).

Due to its widespread adoption and scientific nature, Dairy Herd Improvement (DHI) data has emerged as the primary method and tool for scientifically managing cattle herds in various developed dairy-producing countries, including Canada, the United States, the Netherlands, Sweden, Japan, and others (10). When the SCCs exceeds 100,000 cells/mL, it can serve as a warning indicator for subclinical mastitis (11). To augment the robustness and early prediction capability of subclinical mastitis diagnosis, this study proposes the incorporation of predictive factors unrelated to SCC, such as milk quality parameters, along with the integration of longitudinal monitoring of SCC (12). This methodology not only enhances the diagnostic capabilities but also serves as an optimal data input channel for machine learning systems. Specifically, it contributes to the creation of models focused on the timely identification of subclinical mastitis.

In recent years, the agricultural landscape has witnessed a rapid evolution in information technology, leading to the mainstream adoption of big data on farms. Numerous studies have explored diverse methods and applications aimed at developing predictive and prescriptive decision support tools. Researcher Nazira Mammadova and colleagues (13) proposed a classification prediction method for somatic cell count (SCC) in dairy cows based on Support Vector Machines. Baştan A et al. (14) conducted a study involving 439 pregnant cows, employing multivariate linear regression analysis and a backward stepwise method to predict the incidence of mastitis. Zhou X et al. (15) utilized eight machine learning algorithms to forecast mastitis in dairy cows, based on 14-dimensional data obtained from automatic monitoring systems and milking systems. They validated the consistency of these variables as predictors of cow diseases within automatic system monitoring. However, these methods may only forecast using data from the few days preceding the disease’s onset, and there is no significant disparity in precision and predictive worth among various models (8). Additionally, certain methods require distinct chemical identification, posing challenges for implementation on most dairy farms and limiting their potential as universally applicable predictive models.

Hence, the primary objective of this study is to develop a precise regression prediction model using neural networks, grounded in monthly Dairy Herd Improvement (DHI) data. However, given the nonlinear gradient optimization nature inherent in neural network regression prediction, challenges such as encountering local minima and the influence of randomness in initial weights and thresholds during training may surface. To address these issues, intelligent optimization algorithms have demonstrated commendable efficacy (16).

The Snake Optimization Algorithm (SO), introduced by Hashim F A et al., is a recent advancement in the domain of intelligent optimization (17). Inspired by the foraging behavior of snakes, this algorithm simulates the path selection and movement strategies of snakes. It has been applied to optimization problems such as predicting non-stationary channels (18), ship radiation denoising (19), and multidimensional microgrid energy management (20). The Snake Optimization Algorithm exhibits excellent efficacy in managing continuous nonlinear problems, offering an innovative approach to tackling the nonlinear gradient optimization issues in neural networks. Nevertheless, obstacles remain, such as disparities in the ability to explore and exploit, as well as limitations like sluggish convergence rate and mediocre optimization precision (18).

Based on the research findings mentioned above, this study proposes a novel regression prediction model, called TBESO-BP, for estimating SCC in dairy cows. TBESO-BP model based on TBESO (Multi-strategy Boosted Snake Optimizer) optimized BP neural network to offer a more efficient prognostic instrument for the prevention and management of subclinical mastitis in dairy cows. Furthermore, this study aspires to be a valuable reference and source of inspiration for addressing regression prediction challenges in diverse fields.

## 2 Materials and methods

In tackling the prediction and diagnosis challenges associated with mastitis in dairy cows, this study implemented a systematic approach to data collection and analysis. The subsequent sections outline the data sources, data processing methods, and approaches employed in establishing predictive models. Through the utilization of these data and methods, the objective is to furnish a more effective and accurate tool for predicting mastitis in dairy cows.

### 2.1 Data source

The experimental data utilized in this study originated from a dairy farm in Gansu Province, China, encompassing all lactating cows on the farm. The dairy farm is equipped with modern agricultural data collection facilities, ensuring comprehensive and meticulously maintained data. These facilities provide optimal conditions for conducting numerical analysis of Dairy Herd Improvement (DHI).

The experiment spans from January 2022 to July 2022, with a sampling frequency of once per month. The dataset comprises 25,155 DHI measurement records from 4,015 lactating cows on the farm. This judicious sampling frequency ensures accuracy while avoiding the complexity of frequent sampling, thereby preserving predictive accuracy (21).

### 2.2 Data processing

Initially, data cleaning was conducted on the original 25,155 Dairy Herd Improvement (DHI) records spanning from January to July 2022. Adhering to the criteria defined in the Chinese Holstein cattle production performance determination technical specification (NY/T. 1450–2007) for abnormal data, entries were removed based on criteria such as fat percentage > 7% or < 2%, protein percentage > 5% or < 2%, 305-day milk yield <2,000 kg, lactation days <5 days, lactation days >405 days, and data without records of calving. Subsequently, preliminary feature selection involved eliminating redundant columns like ‘subtotal’ and ‘average with total,’ addressing missing values, and removing redundant feature columns and empty rows, including ‘calving date,’ ‘calving interval (days),’ ‘group number,’ ‘birth date,’ and ‘sampling date,’ while ensuring data integrity through backup.

Following the initial data selection, 24,835 valid records were obtained. These records include time-related indicators such as “Month” and other features like “Parity (calves),” “Lactation days (days),” and “Milk yield (Kg).” A correlation analysis was performed using a correlation heatmap, leading to the removal of features with high correlations (correlation coefficient > 0.66) (22), such as “305 milk yield (Kg),” “WHI,” and “Total fat (%).” All accessible data and code used for data analysis have been uploaded in the GitHub repository.

### 2.3 Predictor features and target variable

With the data now prepared, a model was constructed to explore the relationships between these selected features and the target variable. This model aims to quantify the predictive impact of each feature, incorporating temporal aspects to capture potential temporal effects. The target variable is the SCC of the current month. During model training and evaluation, the predicted values for the month will be compared with the actual values to validate the model parameters and its performance. The following sections detail the methodology for model evaluation and criteria for assessing prediction accuracy. The finalized selection of features and the target variable for prediction are outlined in Table 1. The dataset contains 19,591 remaining valid records.

**Table 1:** The selected temporal features, predictor features and target variable.

Temporal features^1^	Predictor features	Target variable
Month	Parity (Litter)	Current SCC (10^4/mL)^4^
Precursor SCC (10^4/mL)^2^	Lactation days (days)	
Precursor SCS^3^	Milk yield (kg)	
	Butterfat percentage (%)	
	Protein content (%)	
	Urea nitrogen (mg/dL)	
	Endurance	
	Peak day (days)	
	Lactose	

1 Temporal features are predictive features related to time series in regression prediction.

2 Precursor SCC represents the SCC from the previous month.

3 Precursor SCS represents the somatic cell score from the previous month. Somatic Cell Score (SCS) is a logarithmic transformation of the somatic cell count (SCC). Higher SCS values indicate poorer udder health and potential mastitis.

4 Current SCC (10^4/mL) is the target variable representing the somatic cell count. The model compares predicted and actual SCC values for each month.

### 2.4 BP neural network for regression prediction

For accurate regression predictions in subclinical mastitis diagnosis, a robust modeling approach is essential. This study explores the use of a Backpropagation (BP) neural network, a nonlinear regression model capable of capturing complex relationships between variables. The BP neural network model consists of an input layer, a hidden layer, and an output layer. For each layer in the neural network, the input is first weighted, a bias is added, and then passed through an activation function to obtain the output. The detailed explanation and mathematical formulations are provided in the Supplementary materials.

When the input data *X* is an *n* × *m* matrix, where *n* is the number of data samples and *m* is the number of features for each sample, the network computes a predicted output $Y=\left[{\hat{y}}_{1},{\hat{y}}_{2},\ldots ,{\hat{y}}_{i},\ldots ,{\hat{y}}_{n}\right]$. Choose a $x=\left[x_{1},x_{2},\ldots ,x_{j},\ldots ,x_{m}\right],j=1,2,\ldots ,m$, to calculate its predicted output ${\hat{y}}_{i}$ as Equation 1:


(1) $${\hat{y}}_{i}=g\left(\overset{s}{\underset{k=1}{\sum }}w_{ki}\cdot f\left(\overset{m}{\underset{j=1}{\sum }}w_{jk}x_{j}+b_{k}\right)+b_{i}\right),k=1,2,\ldots ,s$$

Where ${\hat{y}}_{i}$ is predicted output for i-th sample, $x_{j}$ is the j-th features for i-th sample, $w_{ki}$ is the weight between the k-th hidden neuron and the i-th output neuron, $w_{jk}$ is the weight between the j-th feature and the k-th hidden neuron, $b_{k}$ is the bias term for the k-th hidden neuron, $b_{i}$ is the bias term for the i-th output layer neuron, s represents the number of hidden neurons and $g\left(\cdot \right)$, $f\left(\cdot \right)$ are the non-linear activation function, see details in the Supplementary Equations 1–4.

To minimize the prediction error, the BP adjusts the weights during training based on a loss function, the study chooses the Mean Squared Error (MSE) as the loss function as Equation 2:


(2) $$\mathrm{Loss}\left(y_{i},{\hat{y}}_{i}\right)=MSE=\frac{1}{n}\overset{n}{\underset{i=1}{\sum }}{\left(y_{i}-{\hat{y}}_{i}\right)}^{2}$$

where $y_{i}-{\hat{y}}_{i}$ represents the difference between the true value and the predicted value on the test set for a specific data sample.

The gradient descent method is commonly used by updating network parameters based on the gradient information of the error function, gradually reducing the error. But gradient descent can converge slowly if the rate is too small or overshoot if it’s too large. In this study, to better adapt and fit Dairy Herd Improvement (DHI) data and to improve the accuracy of the prediction model, an enhanced optimization algorithm is utilized to replace the gradient descent method of selecting the weights and bias of the BP neural network. In other words, transforming the issue of selecting the neural network’s weights and thresholds into an optimization problem. That means the higher the fitness value obtained by the optimization algorithm, the smaller the corresponding loss function value, resulting in a better prediction as Equation 3:


(3) $$\mathrm{fitness}\left(X_{i}\right)=-\mathrm{Loss}\left(y_{i},{\hat{y}}_{i}\right)$$

### 2.5 Model evaluation criteria

The prediction model is evaluated in terms of both accuracy and error to assess its performance. The standard evaluation criteria are outlined in Table 2, as follows:

**Table 2:** The standard evaluation criteria and their formulas.

Evaluation criteria	Formula
Root Mean Squared Error (RMSE)	$\sqrt{\frac{1}{n}\overset{n}{\underset{i=1}{\sum }}{\left(y_{i}-{\hat{y}}_{i}\right)}^{2}}$
Mean Absolute Error (MAE)	$\frac{1}{n}\overset{n}{\underset{i=1}{\sum }}|y_{i}-{\hat{y}}_{i}|$
Mean Absolute Percentage Error (MAPE)	$\frac{1}{n}\overset{n}{\underset{i=1}{\sum }}\frac{\left|y_{i}-{\hat{y}}_{i}\right|}{\left|y_{i}\right|}$
R-squared ($R^{2}$)	$1-\frac{\overset{n}{\underset{i=1}{\sum }}{\left(y_{i}-{\hat{y}}_{i}\right)}^{2}}{\overset{n}{\underset{i=1}{\sum }}{\left(y_{i}-{\bar{y}}_{i}\right)}^{2}}$

$y_{i}-{\hat{y}}_{i}$ represents the difference between the true value and the predicted value on the test set for a specific data sample.

The initial four indicators serve to quantify prediction errors. Mean Absolute Error (MAE) represents the average of absolute errors, Root Mean Squared Error (RMSE) assesses the deviation between observed and true values and Mean Absolute Percentage Error (MAPE) measures the expected value of relative error loss. *R*^2^ evaluates the percentage of variability in the target variable explained by the model’s explanatory variables, with a higher value indicating better performance, approaching 1.

## 3 Proposed TBESO-BP model

To ensure the scientific validity of the experimental results, the final outcomes were averaged over 10 independent runs. The experiments were conducted in a Windows 11 environment with an Intel(R) Core (TM) i9-14900K CPU running at 3.20GHz, 64.0GB of memory, and all model codes were implemented using MATLAB R2024a, and the following model comparison chart corresponds to this configuration.

### 3.1 Comparing optimization algorithms in BP

For this study, comparative experiments were conducted to evaluate the following three BP model variations: the classic BP model (23), AHL-BP (Improved BP with Auto Hiding Learning) (24), PSO-BP (Particle Swarm Optimization-based BP model) (25), and the SO-BP (Snake Optimizer Generalized Regression Neural Network model) (17).

In the comparative experiments, the population size N was set to 100, and the maximum number of iterations was set to 50 for both PSO-BP and SO-BP, as these are population-based swarm intelligence optimization algorithms. The population size N represents the total number of individuals in the population, which plays a key role in balancing exploration and exploitation. Therefore, N=100 was chosen to balance computational efficiency with maintaining sufficient diversity to avoid local optima, based on experimentation and the complexity of the problem. The lower boundary LBand upper boundary UB were set to −1 and 1, respectively.

**Figure 1:**
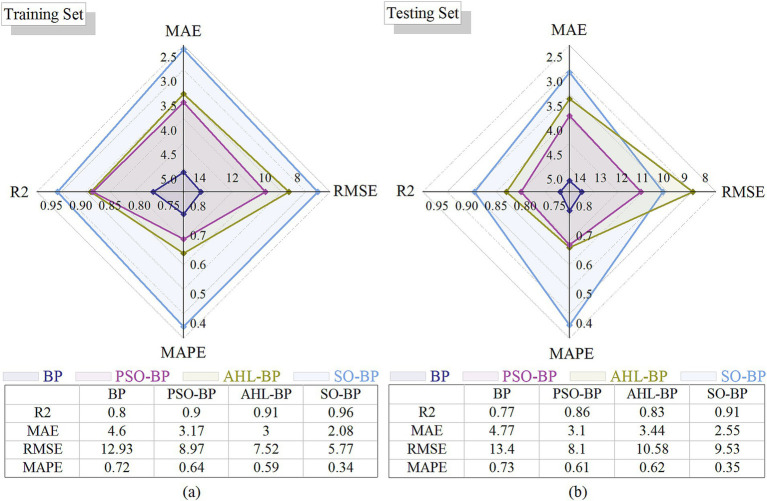
Radar charts of performance comparison **(a)** Training set; **(b)** Testing set.

The radar chart below compares the performance of these four models on the training and testing datasets using RMSE, MAPE, MAE, and R^2^ metrics in Figure 1. Here, lower values for RMSE, MAPE, and MAE indicate better model accuracy, while higher *R*^2^ values signify better fit quality. In this radar chart, each axis represents a different evaluation metric, with larger areas indicating superior overall performance.

As shown in Figure 1, the SO-BP model has the largest area, thus outperforming the other models, which is why the snake optimization algorithm (SO) was selected as the primary improvement approach.

SO effectively tackles single-objective optimization by adjusting its global exploration and local exploitation phases based on the iteration count (17), but its initial exploration focus can lower convergence rates, and later exploitation may risk local optima trapping.

### 3.2 Improved strategies within the TBESO

To address these limitations, The Multi-strategy Boosted Snake Optimizer (TBESO) for enhanced prediction accuracy of subclinical mastitis in cows incorporates Tent Chaotic Mapping (TCM), Bidirectional Search (BDS), and Elite Reverse Learning (EOBL), enhancing SO’s adaptability for complex optimization challenges.

The TBESO algorithm enhances performance in four stages (3.2.1–3.2.4). Firstly, in the initialization stage (3.2.1), Tent chaotic mapping (TCM) is introduced to generate the initial population, increasing population diversity. In the exploration stage (3.2.2), Bidirectional Search (BDS) is employed to update foraging positions, accelerating the convergence of TBESO. In the exploitation stage (3.2.3), the snake population updates individual positions through fighting and mating. Finally, in the population updating stage (3.2.4), the Elite reverse learning (EOBL) mechanism is introduced to further optimize the population, expanding the search range of TBESO and preventing it from getting trapped in local optima.

#### 3.2.1 Population initialization (introducing Tent chaotic mapping)

TBESO uses a chaotic mapping approach (26) in this step, unlike the original Snake Optimization algorithm. This random and convenient technique preserves population diversity. It helps Snake Optimization avoid local traps and explore globally. Random snakes are dispersed uniformly.

The definition of the Tent chaotic mapping algorithm is as Equation 4:


(4) $$X_{i}\left(t+1\right)=f\left(X_{i}\left(t\right)\right)=\{\begin{array}{c} \alpha X_{i}\left(t\right),\;\mathrm{if}\;x<0.5\\ \alpha \left(1-X_{i}\left(t\right)\right),\;\mathrm{if}\;x>0.5 \end{array}$$

Here, $X_{i}$ represents the position of the i-th snake at iteration t, $X_{i}\left(t+1\right)$ is the updated position of the i-th snake at the next iteration, α represents a threshold parameter $\left(0\leq \alpha \leq 2\right)$ that divides the function into two conditional segments, here takes a value of 1.999, and t is the iteration step, representing the number of updates applied to the position.

**Figure 2:**
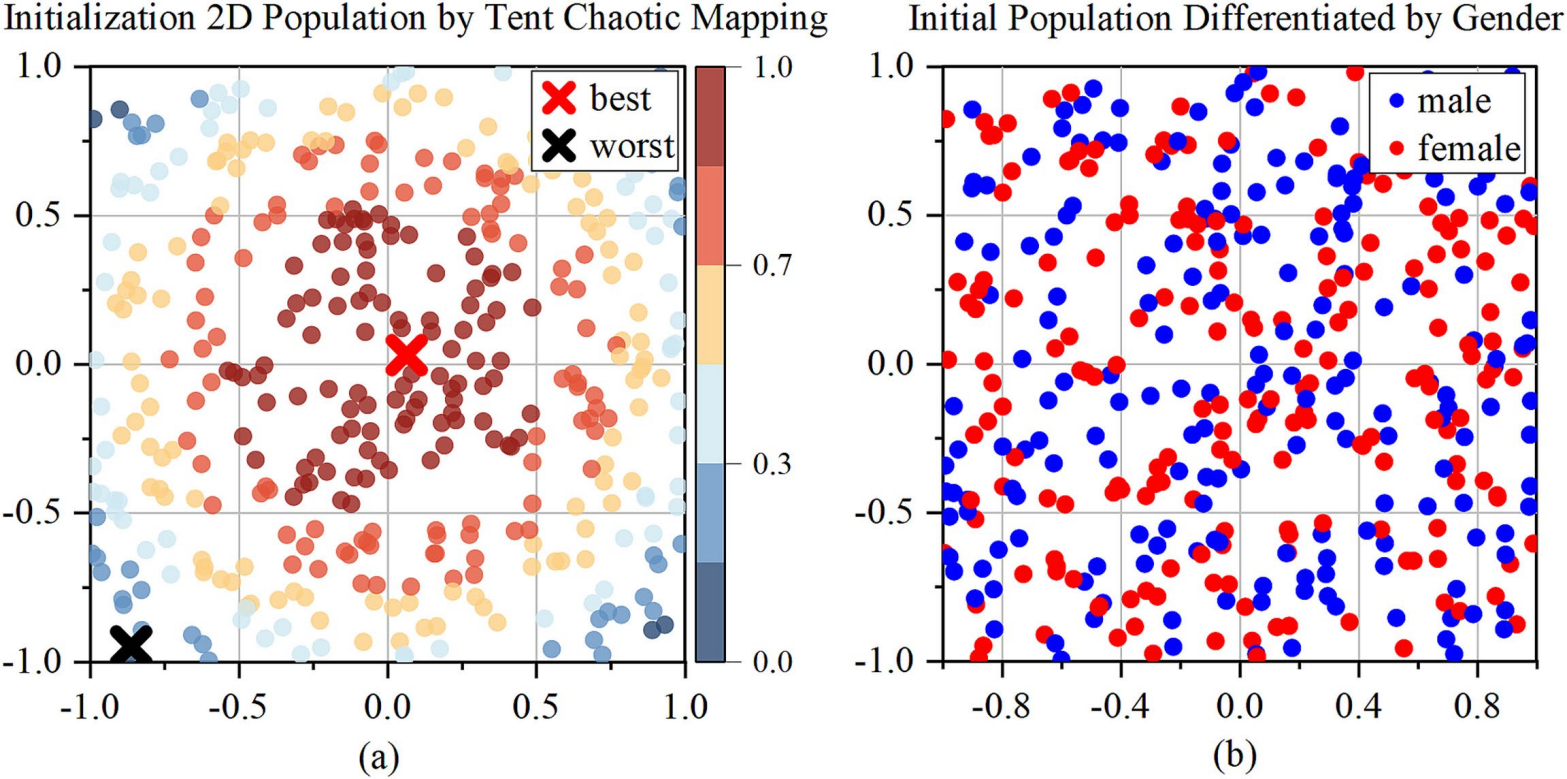
Initialization of 2D population by Tent chaotic mapping. **(a)** 2D initialization population; **(b)** Initial population differentiated by gender.

The Tent chaotic mapping generates a uniformly distributed population, with a population size of N. After generating the initial population, the population is divided into male and female groups, with $N_{m}$ representing the number of males and $N_{f}$ representing the number of females as Figure 2b.

To demonstrate the evolution of candidate solutions over the iterations, the study visualizes the population distribution in Supplementary materials. The population distribution of the TBESO is examined after 25 iterations in a two-dimensional space. The population size (N) is set to 400, with the upper boundary (UB) and lower boundary (LB) defined as 1 and −1, respectively and the following population distribution chart corresponds to this configuration. Additionally, an ablation experiment using the CEC 2017 benchmark suite is conducted to assess the effectiveness of the TBESO algorithm under various conditions. Specific details of this ablation experiment can be found in the Supplementary materials.

In Figure 2a, which depicts the initial population generated by Tent chaotic mapping, the blue points are farther from the origin. Figure 2b distinguishes male and female populations using red and blue points.

#### 3.2.2 Exploration stage (introducing bidirectional search)

The food quality Q determines whether the snake population enters the foraging stage (exploration stage) or the mating and reproduction mode (exploitation stage). Additionally, Snake behavior is also affected by temperature when food is abundant.

Defining Food quantity Q can be obtained using the Equation 5:


(5) $$Q=c_{1}*\exp\left(\frac{t-T}{T}\right)$$

Where t refers to the current iteration and T refers to the maximum number iterations. The constant $c_{1}$ is set to a specific value, in this case, $c_{1}=0.5$.

If Q<Threshold (Threshold = 0.25), indicating insufficient food, the snake population will update its positions through two methods. One is random updating, which is similar to the original Snake Optimization algorithm. The other is adopting a bidirectional search strategy. The position update will choose the method that produces a higher fitness value between the two.

##### 3.2.2.1 Random updating

Random updating as Equations 6, 7:


(6) $$X_{i}^{m}\left(t+1\right)=X_{\mathrm{rand}}^{m}\left(t\right)\pm c_{2}\times A_{m}\times \left(\left(ub-\mathrm{lb}\right)\times \mathrm{rand}+\mathrm{lb}\right)$$

(7) $$X_{i}^{f}\left(t+1\right)=X_{\mathrm{rand}}^{f}\left(t\right)\pm c_{2}\times A_{f}\times \left(\left(ub-\mathrm{lb}\right)\times \mathrm{rand}+\mathrm{lb}\right)$$

In the provided context, $X_{i}^{m}$ and $X_{i}^{f}$ denote the randomly chosen positions of male and female individuals, respectively, where rand is a random scalar generated from a uniform distribution in the range (0,1). The constant $c_{2}$ is a constant and equals 0.05, and ub, lb are the lower and upper bounds of the problem, respectively. The ± symbol represents the flag direction operator (diversity factor), which randomly chooses between increasing or decreasing the solution’s position, enabling more effective exploration of the solution space. Additionally, $A_{m}$ and $A_{f}$ represent the ability of males and females to search for food, and they can be calculated as Equations 8, 9:


(8) $$A_{m}=\exp\left(-\frac{\mathrm{fitness}\left(X_{\mathrm{rand}}^{m}\left(t\right)\right)}{\mathrm{fitness}\left(X_{i}^{m}\left(t\right)\right)}\right)$$

(9) $$A_{f}=\exp\left(-\frac{\mathrm{fitness}\left(X_{\mathrm{rand}}^{f}\left(t\right)\right)}{\mathrm{fitness}\left(X_{i}^{f}\left(t\right)\right)}\right)$$

Where $\mathrm{fitness}\left(X_{\mathrm{rand}}^{m}\left(t\right)\right)$ and $\mathrm{fitness}\left(X_{\mathrm{rand}}^{f}\left(t\right)\right)$ are the fitness of $X_{\mathrm{rand}}^{m}$ and $X_{\mathrm{rand}}^{f}$ of rand-th individuals at iteration t in male and female group, respectively.

##### 3.2.2.2 Bidirectional search strategy

**Figure 3:**
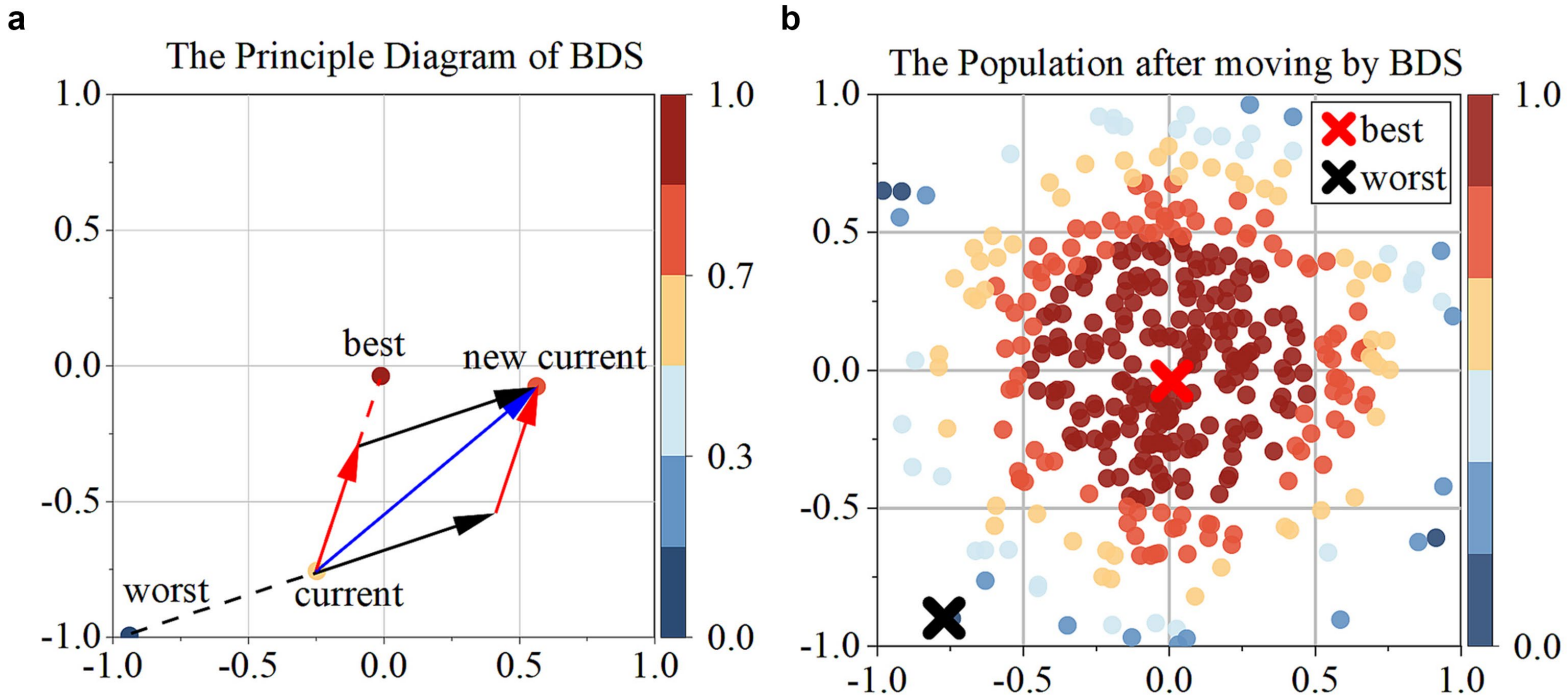
**(a)** Presents the principal diagram of BDS; **(b)** the population after moving by BDS.

The bidirectional search algorithm simultaneously initiates two independent searches, with one progressing forward from the start and the other moving backward from the target. The principle of Bidirectional search is to guide individuals iteratively, moving them away from the worst individual while simultaneously approaching the best individual (Figure 3).

In this context, deep blue individuals represent the worst individual ($d_{\mathrm{worst}}$), deep red individuals represent the best individual ($d_{\mathrm{best}}$), black arrows represent the vector ($\vec{F_{\mathrm{worst}}})$ moving away from the worst individual, with the Equations 10, 11:


(10) $$\begin{array}{l} X_{i}^{m}\left(t+1\right)=X_{i}^{m}\left(t\right)+\mathrm{ran}d_{1}\times \left(X_{\mathrm{best}}^{m}-X_{i}^{m}\left(t\right)\right)-\\ \mathrm{ran}d_{2}\times \left(X_{\mathrm{worst}}^{m}-X_{i}^{m}\left(t\right)\right) \end{array}$$

(11) $$\begin{array}{l} X_{i}^{f}\left(t+1\right)=X_{i}^{f}\left(t\right)+\mathrm{ran}d_{1}\times \left(X_{\mathrm{best}}^{f}-X_{i}^{f}\left(t\right)\right)-\\ \mathrm{ran}d_{2}\times \left(X_{\mathrm{worst}}^{f}-X_{i}^{f}\left(t\right)\right) \end{array}$$

Where $X_{\mathrm{best}}^{f}$ and $X_{\mathrm{worst}}^{f}$ represent the best and worst female individuals, and $X_{\mathrm{best}}^{m}$ and $X_{\mathrm{worst}}^{m}$ represent the best and worst male individuals. $\mathrm{ran}d_{1}$ and $\mathrm{ran}d_{2}$ are two randomly generated random numbers from uniform distribution in the interval (0, 1).

#### 3.2.3 Exploitation stage

If Q>Threshold (Threshold = 0.25) represents sufficient food. In the presence of abundant food, the behaviors of the snake population are influenced by temperature. When the temperature is high and food is plentiful, the snake population will move toward food and consume the existing resources. However, only when the temperature is low and there is sufficient food will be mating, and reproduction occur.

The Temperature Temp can be defined using the Equation 12:

(12) $$\mathrm{Temp}=\exp\left(\frac{-t}{T}\right)$$

1. When Temp>0.6, indicating high temperature, the snake population will move to the food only as Equation 13:

(13) $$X_{i}\left(t+1\right)=X_{\mathrm{best}}\pm c_{3}\times \mathrm{Temp}\times \mathrm{rand}\times \left(X_{\mathrm{best}}-X_{i}\left(t\right)\right)$$

Where $X_{i}$ is the position of individual (male or female), $X_{\mathrm{best}}$ is the position of the best individuals among $X_{\mathrm{best}}^{m}$ and $X_{\mathrm{best}}^{m}$, which is also the best one in the whole population, and guides the entire population during the optimization process, and $c_{3}=2$.

2. When Temp<0.6, in low temperature, the snake will be in the fight mode or mating mode randomly. The probability of choosing fighting mode is when rand>0.6, while mating mode is selected when rand≤0.6. Here, rand is a random number drawn from a uniform distribution in the range (0, 1).

In the fighting mode, each male will fight to get the best female, and each female will try to select the best male as Equations 14, 15:


(14) $$X_{i}^{m}\left(t+1\right)=X_{i}^{m}\left(t\right)+c_{3}\times FM\times \mathrm{rand}\times \left(Q\times X_{\mathrm{best}}^{f}-X_{i}^{m}\left(t\right)\right)$$

(15) $$X_{i}^{f}\left(t+1\right)=X_{i}^{f}\left(t\right)+c_{3}\times FF\times \mathrm{rand}\times \left(Q\times X_{\mathrm{best}}^{m}-X_{i}^{f}\left(t\right)\right)$$

Where $X_{i}^{m},X_{i}^{f}$ refers toi-th male position, $X_{\mathrm{best}}^{f},X_{\mathrm{best}}^{m}$ refers to the positions of the best individual in female group, and FM,FF are the fighting ability of male, female agents, respectively. FM and FF can be calculated from the Equations 16, 17:


(16) $$FM=\exp\left(-\frac{\mathrm{fitness}\left(X_{\mathrm{best}}^{f}\right)}{\mathrm{fitness}\left(X_{i}^{m}\left(t\right)\right)}\right)$$

(17) $$FF=\exp\left(-\frac{\mathrm{fitness}\left(X_{\mathrm{best}}^{m}\right)}{\mathrm{fitness}\left(X_{i}^{f}\left(t\right)\right)}\right)$$

In the mating mode, the mating occurs between each pair related to the availability of food quantity Equations 18, 19:


(18) $$\begin{array}{l} X_{i}^{m}\left(t+1\right)=X_{i}^{m}\left(t\right)+c_{3}\times MM\times \mathrm{rand}\times \\ \left(Q\times X_{i}^{f}\left(t\right)-X_{i}^{m}\left(t\right)\right) \end{array}$$

(19) $$X_{i}^{f}\left(t+1\right)=X_{i}^{f}\left(t\right)+c_{3}\times MF\times \mathrm{rand}\times \left(Q\times X_{i}^{m}\left(t\right)-X_{i}^{f}\left(t\right)\right)$$

Where $X_{i}^{m},X_{i}^{f}$ are the position of i-th agent in male and female group and MM,MF refers to the mating ability of male and female, respectively, and they can be calculated as Equations 20, 21:


(20) $$MM=\exp\left(-\frac{\mathrm{fitness}\left(X_{i}^{f}\left(t\right)\right)}{\mathrm{fitness}\left(X_{i}^{m}\left(t\right)\right)}\right)$$

(21) $$MF=\exp\left(-\frac{\mathrm{fitness}\left(X_{i}^{m}\left(t\right)\right)}{\mathrm{fitness}\left(X_{i}^{f}\left(t\right)\right)}\right)$$

During the mating process in the search space, a female snake may lay eggs. The egg (in the pseudocode) represents a randomly selected value of either 1 or −1 and simulates the process of laying eggs (generating new solutions) and hatching (replacing poor solutions with potentially better ones), thereby promoting exploration of the search space. If egg equals 1, select worst male and female and replace them as Equations 22, 23:


(22) $$X_{\mathrm{worst}}^{m}=\mathrm{lb}+\mathrm{rand}\times \left(ub-\mathrm{lb}\right)$$

(23) $$X_{\mathrm{worst}}^{f}=\mathrm{lb}+\mathrm{rand}\times \left(ub-\mathrm{lb}\right)$$

Where $X_{\mathrm{worst}}^{m},X_{\mathrm{worst}}^{f}$ are the worst individuals in male and female group. Here, ub, lb are the lower and upper bounds of the problem, respectively.

#### 3.2.4 Population update (introduction of Elite Reverse Learning)

**Figure 4:**
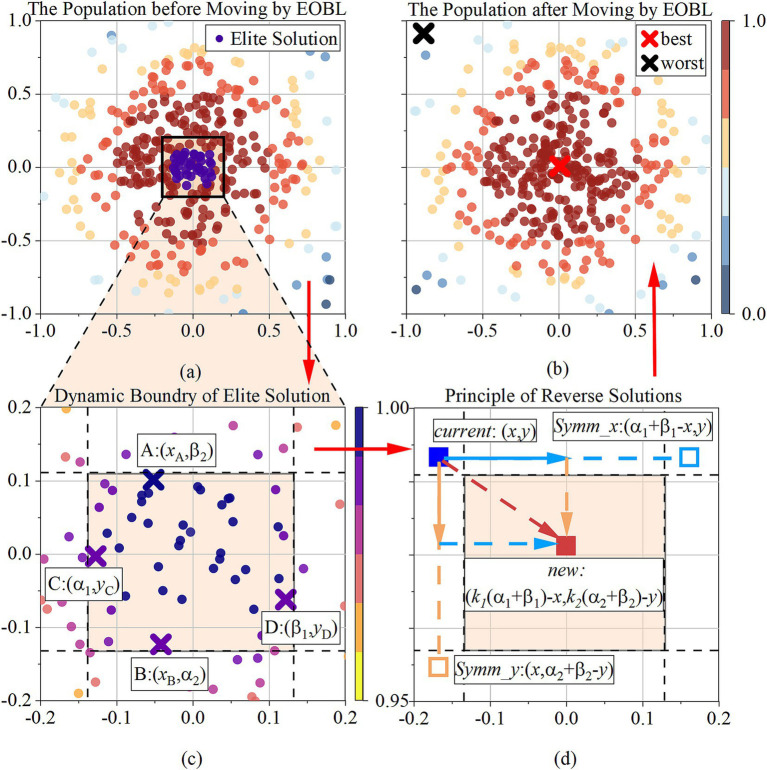
**(a)** The population before moving by EOBL; **(b)** the population before moving by EOBL; **(c)** presents how the dynamic boundary of elite solution is determined; **(d)** presents how the reverse solution is determined; The sequence is **(a)**, **(c)**, **(d)**, **(b)**, along the red arrow.

After the exploitation stage, to address the issue of poor local development capability in the Snake Optimization algorithm, TBESO introduces the Elite Reverse Learning strategy (27). This strategy leverages the characteristic that elite individuals contain more effective information than general individuals. It creates a reverse population using elite individuals to increase population diversity. The best individual from the new population is then selected for the next generation iteration, utilizing the neighborhood space of elite individuals and enhancing local development capability, as Figure 4.

The elite solution is the best solution in the current population based on their fitness values, depicted as the colored square in the Figures 4a,c. Its definition as $X_{p,j}^{e}=\left\{X_{p,j}^{e},X_{p,j}^{e},\cdots ,X_{p,d}^{e}\right\}$, where p represents the index of each elite solution within the group of elites, and p=1,2,…,s. s is the total number of elite solutions. The study determined it as s=0.1×N, which N is the number of populations. d represents the specific dimension within an elite solution vector, indexed by j=1,2,…,d, where d is the total number of dimensions.

Given a current solution current represents the i-th individual in Figure 4d, where $\alpha_{j}$ and $\beta_{j}$ denote the dynamic boundaries in the elite group, representing the maximum and minimum values in dimension j, expressed as $\alpha_{j}=\min\left(X_{p,j}^{e}\right)$, $\beta_{j}=\max\left(X_{p,j}^{e}\right)$. The elite reverse solution of the i-th individual $X_{i,j}$ is new: $OP_{i}=\left\{OP_{i,1},OP_{i,2},\cdots ,OP_{i,j}\right\}$ in Figure 4d, as Equation 24:


(24) $$OP_{i,j}=\mathrm{rand}\cdot \left(\alpha_{j}+\beta_{j}\right)-X_{i,j}$$

where rand is a random scalar generated from a uniform distribution in the range (0,1).

Dynamic boundaries overcome the drawback of fixed boundaries in preserving search experience, enabling the reverse solution of elites to be in a narrow and dynamic search space, facilitating algorithm convergence. If the dynamic boundary operation causes $OP_{i,j}^{e}$ to cross the boundary and become an infeasible solution, it can be reset using a randomly generated method, as Equation 25:


(25) $$OP_{i,j}=\mathrm{rand}\cdot \left(\alpha_{j}-\beta_{j}\right)+\beta_{j},\;\mathrm{if}\;OP_{i,j}<lb_{j}\|OP_{i,j}>ub_{j}$$

where rand is a random scalar generated from a uniform distribution in the range (0,1), $lb_{j}$ and $ub_{j}$ represents lower boundary upper boundary for j dimension, respectively.

The calculation updates the current solution within a symmetric range around the elite individual, introducing randomness in both the *x*-axis and *y*-axis directions ($Symm_{x}\;\mathrm{and}\;Symm_{y}$). This promotes diversity in search, helping to explore multiple regions of space and avoid local optima. If the new solution is better, it will replace the current one, ensuring continuous improvement and refinement of the best solutions over iterations. Figure 4b shows the population distribution after being updated by the EOBL.

### 3.3 The proceeding of TBESO

Combining the Tent chaotic mapping, bidirectional search strategy, and elite reverse learning strategy mentioned above, the pseudocode for the TBESO algorithm is as follows. Here, N represents the population size, pos is the initial population generated by the Tent chaotic mapping algorithm, T represents the maximum number of iterations, LB and UB are the lower and upper bounds of the optimization problem, dim is the dimension of the problem, Q represents food quality, Temp represents the current temperature, rand is a random number drawn from a uniform distribution in the range (0, 1), egg represents a randomly selected value of either 1 or −1 (Algorithm 1).

**ALGORITHM 1:**
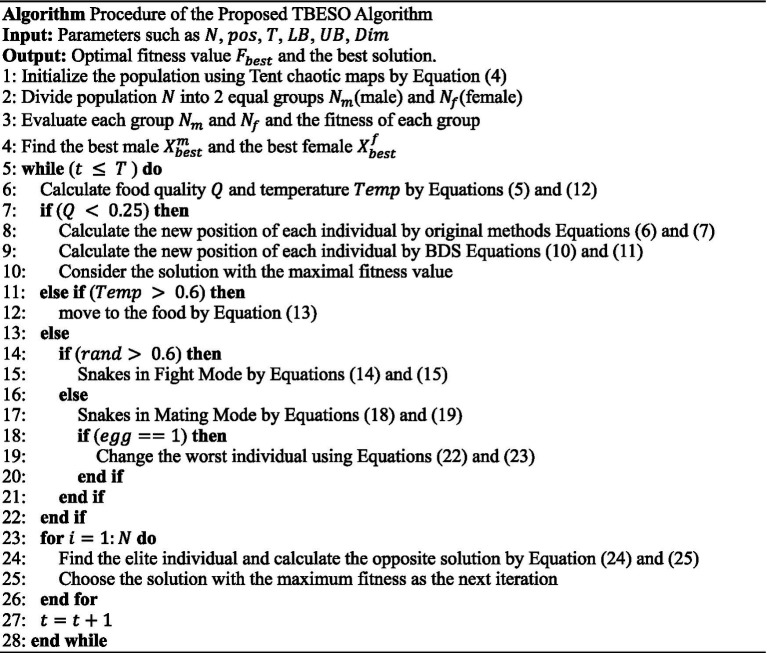
Procedure of the proposed TBESO algorithm.

The pseudocode is as follows:

### 3.4 TBESO-BP

Upon acquiring TBESO, the procedure selects neural network initial weights ($w_{i}$) and thresholds ($b_{i}$). The model compares the total sum of the discrepancies between the anticipated values generated by the neural network and the actual values. This loss represents the TBESO iterative optimization fitness function.

**Figure 5:**
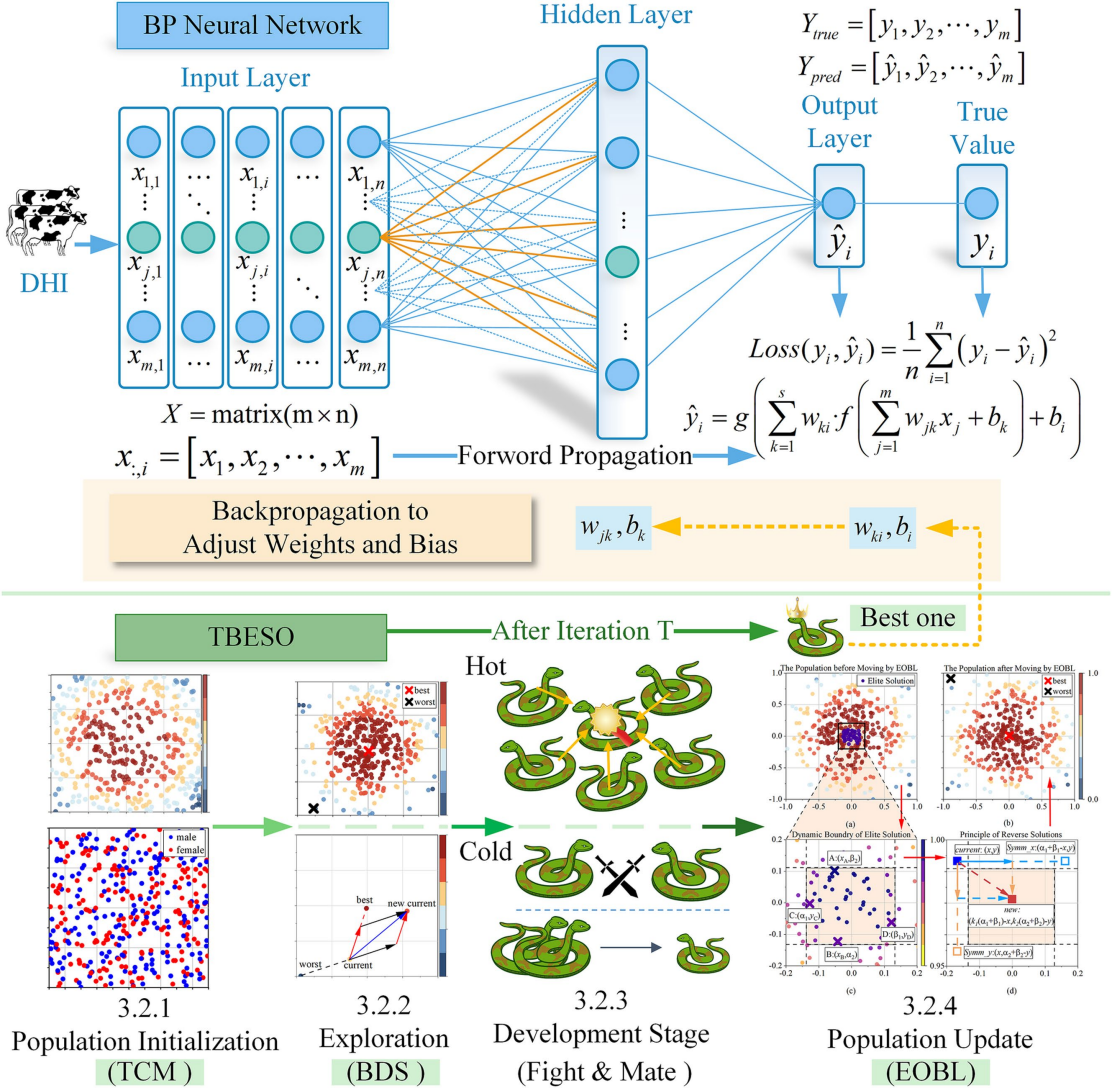
The process of TBESO-BP model.

Once TBESO reaches the defined objective conditions, the related weights and thresholds are regarded as the starting optimal values for the backpropagation (BP) neural network. This method reduces absolute disparities to improve model performance and forecast accuracy. Figure 5 shows that iteratively selecting BP neural network initial weights and thresholds improves model performance.

## 4 Model evaluation and results

To evaluate the SO-BP, BEESO-BP [Improved BP based on Multi-strategy Boosted Snake-Inspired Optimizer (27), which proposed by researchers Gang Hu and Rui Yang], and the TBESO-BP were compared for predicting the number of cow somatic cells, we conducted comparative experiments using the same environment and parameters as previously described. Each model was tested with a population size of 100 and 50 maximum iterations, and results were averaged over 10 independent runs.

### 4.1 Model performance comparison

**Figure 6:**
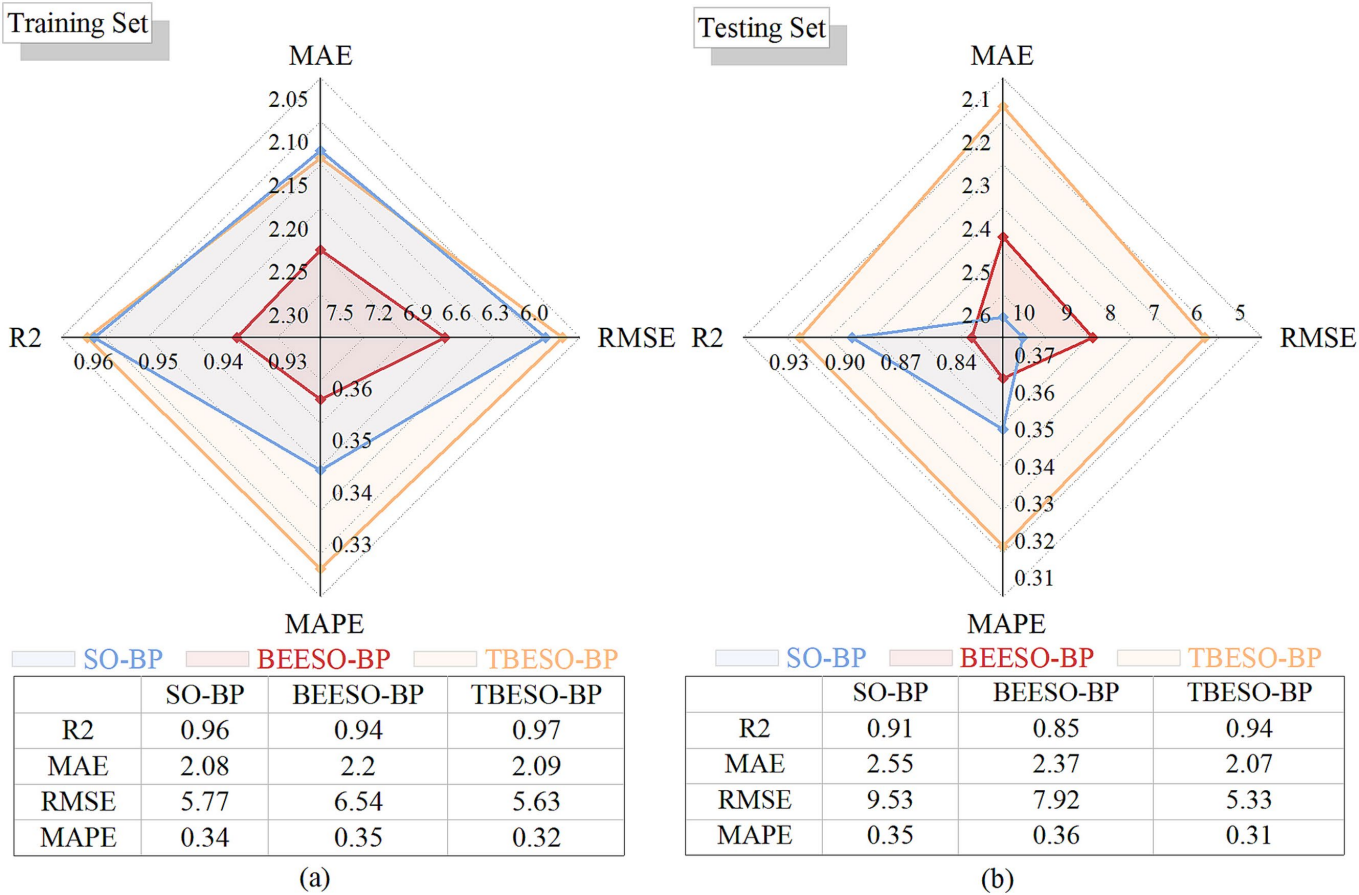
Radar charts of performance comparison. **(a)** Training set; **(b)** Testing set.

These radar charts were used in Figure 6 to compare each model’s results across four performance metrics: R^2^, MAE, RMSE, and MAPE. The radar chart provides an intuitive visualization of model strengths and weaknesses across these axes, highlighting which models performed best in specific areas. A larger area within the radar chart indicates a superior overall performance of the model, as it reflects higher predictive accuracy (R^2^) and lower error metrics (MAE, RMSE, and MAPE).

### 4.2 The results of comparison

Figure 6a shows that in the training set, the TBESO-BP model achieves an R-squared of 96.60%, with MAE, RMSE, and MAPE values of 2.09, 5.63, and 32.05%, respectively slightly outperforming both the SO-BP and BEESO-BP models.

Similarly, Figure 6b illustrates the TBESO-BP model’s performance on the testing set, achieving an R-squared of 93.97% and error metrics of 2.07 (MAE), 5.04 (RMSE), and 31.36% (MAPE), again surpassing the SO-BP and BEESO-BP models.

## 5 Analysis and discussion

To analyze and compare the predictive performance and efficiency of each model, including BP, PSO-BP, AHL-BP, SO-BP, BEESO-BP, and the proposed TBESO-BP, we evaluate performance metrics such as MAE, RMSE, MAPE and R^2^, as shown in Figures 1, 6. These figures allow for an easy comparison of model efficiency and accuracy. Smaller values for MAE, RMSE, and MAPE indicate better performance, while a higher R^2^ suggests improved fitting accuracy. Each of these metrics directly correlates with practical performance, highlighting the strengths and weaknesses of the models in applications.

### 5.1 Models comparison analysis

The comparison results entail the six regression prediction models, with the evaluation of error quantification standards such as MAE, RMSE, MAPE, and the assessment of correctness quantification using R2. The predicted performances of the six models differ and are studied as follows:

#### 5.1.1 Accuracy analysis

Upon evaluating *R*^2^ for accuracy in Figures 1, 6, all six models show *R*^2^ values exceeding 76.57% on both training and test sets, indicating generally robust predictive performance. However, the TBESO-BP model stands out with a test R^2^ of 93.97%, outperforming other models by 22.72, 48.60, 12.86, 3.09, and 10.96% compared to BP, AHL-BP, PSO-BP, SO-BP, and BEESO-BP, respectively. This highlights that the TBESO-BP model demonstrates exceptional accuracy in predicting somatic cell count.

#### 5.1.2 Error analysis

Upon evaluating error quantification standards MAE, RMSE, and MAPE in Figures 1, 6, the TBESO-BP model outperforms the other models, with smaller errors across both the training and test sets. On the test set, TBESO-BP achieves reductions of 56.60% in MAE, 56.46% in RMSE, and 40.29% in MAPE compared to the BP model. Additionally, compared to other models such as AHL-BP, PSO-BP, SO-BP, and BEESO-BP, TBESO-BP demonstrates improvements across all metrics, reducing MAE by 33.23, 40.00, 18.82, and 14.49%, respectively.

Lower MAE in TBESO-BP model indicates that its predictions are closer to actual values, which is crucial for predicting SCC, a key indicator for subclinical mastitis. Accurate predictions of SCC are essential for effective farm management and timely intervention decisions. Furthermore, the smaller MAPE of TBESO-BP highlights its ability to maintain accurate predictions, even in situations with large variations in data ranges. The smaller RMSE highlights the model’s ability to remain consistent and less influenced by a few extreme data samples.

### 5.2 Different models for various applications

**Figure 7:**
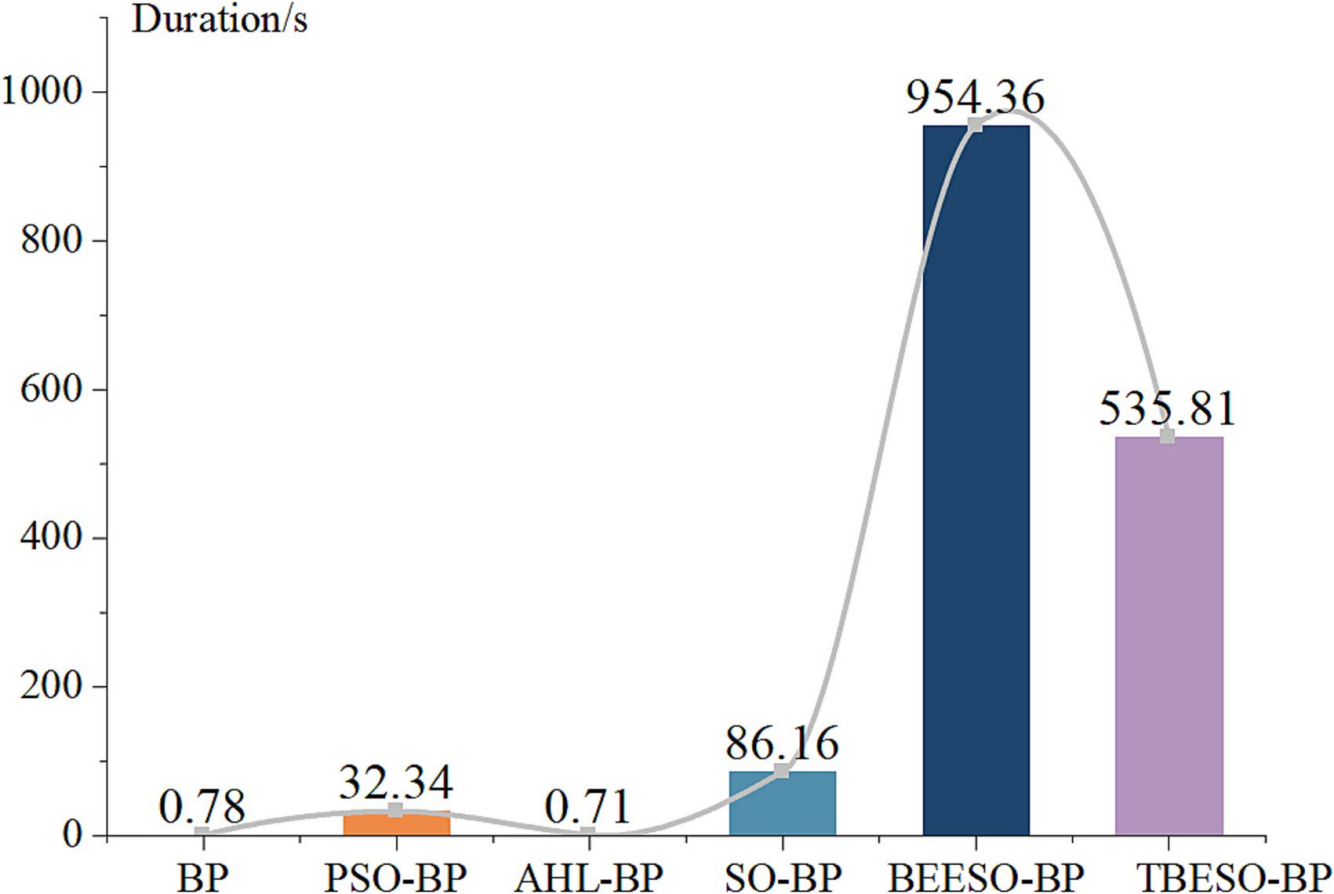
Mean duration comparison plot of 6 models.

When selecting an appropriate prediction model, it is essential to consider not only error metrics like MAE, RMSE, R^2^, and MAPE but also the model’s runtime. In practical applications, the runtime can significantly impact tasks with high real-time demands. Therefore, by analyzing the runtime of each model, we gain further insights into their suitability for different use cases. Figure 7 presents the comparison of the mean duration of the six models as follows:

Figure 7 reveals that the classic BP, AHL-BP, and PSO-BP models have durations all below 33 s, with RMSE values exceeding 8. The lowest R-squared is for BP at 76.57% according to Figures 1b, 6b, while the highest is for AHL-BP at 85.74%. In contrast, the SO-BP, BEESO-BP, and TBESO-BP models have durations exceeding 85 s, with error values all below 100. The TBESO-BP model has the lowest error value at only 30.67 and all three models have R-squared above 84.68%, with TBESO-BP being the highest at 93.97%. This indicates that overall prediction duration increases with improved prediction performance. In practical applications, the choice of the prediction model should be based on the specific usage scenario.

In summary, among the six comparative prediction models, the AHL-BP is better suited for scenarios with lower accuracy requirements and higher real-time demands. For example, in disease warning software, it can provide real-time fuzzy warnings for potential subclinical mastitis in cows when cow information is updated, allowing for timely preventive measures. On the other hand, the TBESO-BP model is better suited for scenarios with higher accuracy requirements and lower real-time demands. For instance, after entering cow information, it can accurately predict SCCs for subclinical mastitis in cows, facilitating a more precise assessment of the severity of the condition.

### 5.3 Disease prediction

**Figure 8:**
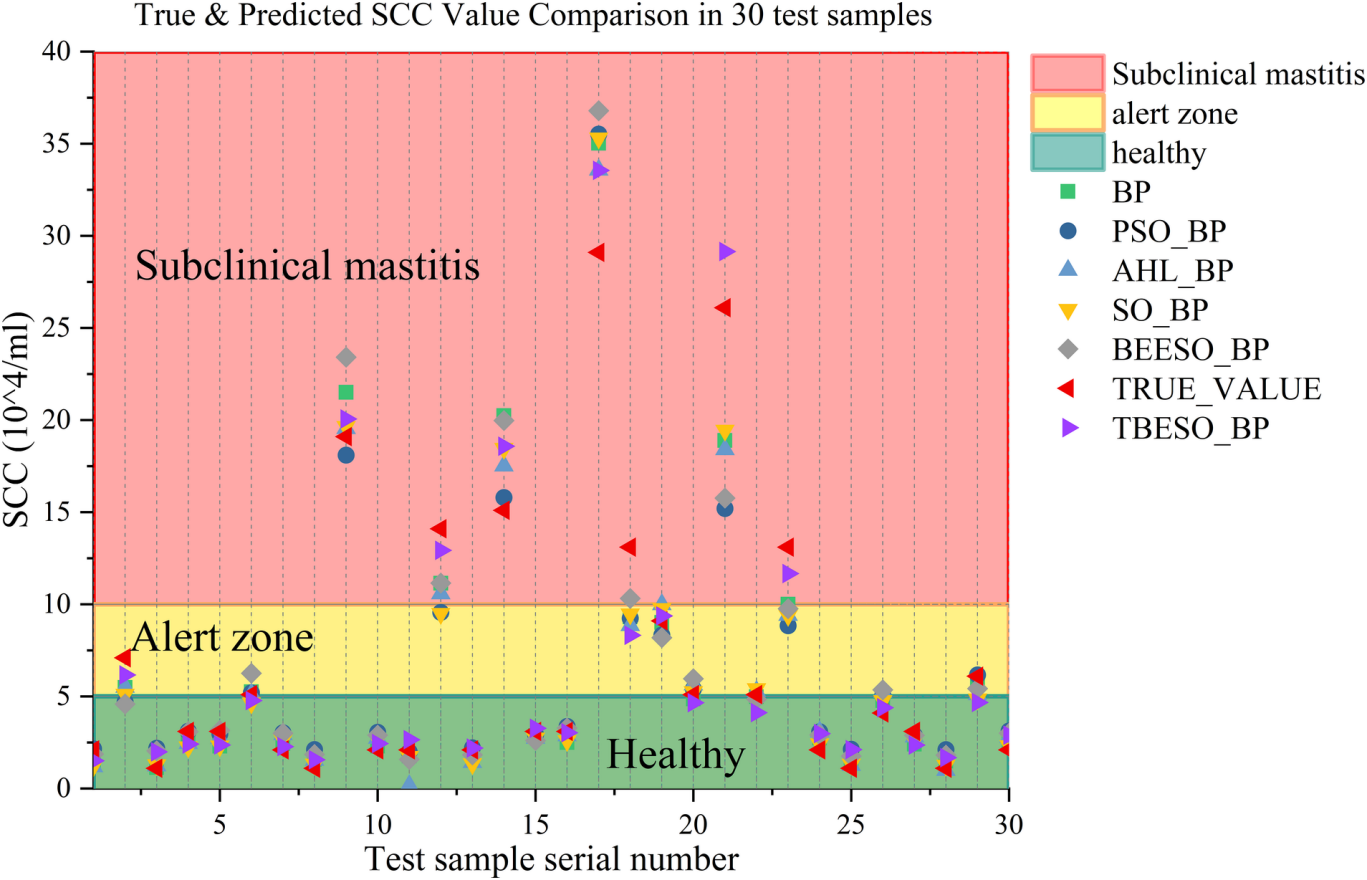
True & predicted SCC value comparison in 30 test samples.

To analyze the models’ predictive accuracy in the context of subclinical mastitis in dairy cows, Figure 8 illustrates the real versus predicted values of somatic cell counts (SCCs) for each of the six models across 30 test samples. The *x*-axis represents the sample sequence, and the *y*-axis shows SCC values in units of 10^4^/ml. The TBESO-BP model’s predictions are shown as a blue line, and the true SCC values are marked in red line. Other models’ predictions are represented by scatter plots for comparison.

In this chart, the colored background conveys health status based on SCC thresholds: green represents the healthy range (SCC ≤ 50,000/ml), yellow indicates a cautionary range, and red marks the subclinical mastitis diagnostic threshold (SCC > 100,000/ml) (28). The chart effectively highlights the TBESO-BP model’s capability to predict SCC values, crucial for accurate early detection and intervention.

Figure 8 demonstrates that the TBESO-BP model’s predictions align more closely with the actual SCC values than those of the other models, highlighting its superior accuracy. Notably, in this sample set, SCC values for samples 9, 12, 14, 17, 21 and 23 fall within the red background area, indicating probably are subclinical mastitis. Samples 2, 6, 19, and 29 are situated in the yellow caution zone, while the remaining samples are within the green, healthy range.

The capabilities of these models enable them to accurately predict SCCs and growth trends in cows, providing real-time health information for breeders and farm managers, which is crucial for subclinical mastitis warning. This holds significant implications:

1. **Early Detection and Prevention**: Through regression prediction models, potential diseased cows can be identified in the early stages of disease development, allowing for timely intervention, improving treatment success rates, and avoiding vaccine wastage.

2. **Precision Warning System**: Utilizing regression prediction models, we can construct a more precise warning system with abundant data. This enables breeders to make more accurate decisions based on individual characteristics and health status of each cow, effectively reducing the incidence and impact of subclinical mastitis.

3. **Resource Optimization**: Regression prediction models assist farm owners or breeders in more efficiently allocating resources. Targeted interventions can prevent unnecessary treatment and additional expenses, optimizing resource utilization while enhancing cow health.

4. **Data-Driven Decision-Making**: By employing regression prediction models, historical and real-time data can better support decision-making in livestock management. This contributes to the scientific and predictable management of livestock, making the industry more stable and sustainable.

5. **Scientific Research and Innovation**: Regression prediction models provide researchers with a high-quality data source for in-depth studies on pathogenesis, influencing factors, and preventive measures of subclinical mastitis in dairy cows. This data-driven approach fosters innovation and development in relevant fields.

In conclusion, this study not only validates the effectiveness of the six prediction models for SCC prediction but also introduces a new and more accurate regression prediction model, TBESO-BP, for health management in dairy farming. This will contribute to improving breeding efficiency, optimizing resource utilization, and enhancing the production and welfare of dairy cows.

## 6 Conclusion

In addressing the challenges of accuracy and quantitative prediction in traditional methods for predicting subclinical mastitis in dairy cows, this study proposes a Body SCC Prediction Model based on Improved Snake Optimization Algorithm and Backpropagation (TBESO-BP). The model incorporates tent chaotic mapping, elite reverse learning, and bidirectional search strategies to enhance the population diversity, convergence speed, and local development capability of the snake optimization algorithm. Using TBESO, the initial weights and thresholds of the BP neural network are optimized, and the predictions of six models are objectively evaluated using five evaluation indicators.

The results demonstrate that the TBESO-BP model proposed in this paper exhibits high accuracy and stability in predicting subclinical mastitis in dairy cows. The optimized TBESO algorithm partially addresses issues such as lack of population diversity and susceptibility to local optima in the original algorithm, making it applicable to other problems.

Furthermore, in practical applications, the choice of a suitable prediction model should align with the specific prediction goals. The six comparative models in this study all demonstrate good prediction accuracy. The BP neural network with adaptive hidden layers is more suitable for real-time fuzzy warnings with lower precision requirements but higher immediacy. On the other hand, the TBESO-BP model, which provides accurate predictions of SCCs, is beneficial for assessing the specific condition of subclinical mastitis in dairy cows, particularly in scenarios where higher accuracy is needed without overly prioritizing immediacy. These research findings not only offer new reference points for the prevention and treatment of subclinical mastitis in dairy cows but also provide an accurate and reliable predictive tool.

## Data Availability

Publicly available datasets were analyzed in this study. This data can be found here: https://github.com/Otherisland/SubclinicalMastitisPrediction-TBESO-BP.

## References

[1] Marques-Bastos SLS Coelho MLV De Sousa Santos IN Moreno DSA Barrias ES De Mendonça JFM. Effects of the natural antimicrobial peptide aureocin A53 on cells of Staphylococcus aureus and *Streptococcus agalactiae* involved in bovine mastitis in the excised teat model. World J Microbiol Biotechnol. (2023) 39:5. doi: 10.1007/s11274-022-03443-w, PMID: 36346468

[2] Bochniarz M Szczubiał M Brodzki P Krakowski L Dąbrowski R. Serum amyloid a as an marker of cow’s mastitis caused by Streptococcus sp. Comp Immunol Microbiol Infect Dis. (2020) 72:101498. doi: 10.1016/j.cimid.2020.101498, PMID: 32505957

[3] Wang L Haq SU Shoaib M He J Guo W Wei X. Subclinical mastitis in small-holder dairy herds of Gansu Province, Northwest China: prevalence, bacterial pathogens, antimicrobial susceptibility, and risk factor analysis. Microorganisms. (2024) 12:2643. doi: 10.3390/microorganisms12122643, PMID: 39770845 PMC11727839

[4] Fernandes L Guimaraes I Noyes NR Caixeta LS Machado VS. Effect of subclinical mastitis detected in the first month of lactation on somatic cell count linear scores, milk yield, fertility, and culling of dairy cows in certified organic herds. J Dairy Sci. (2021) 104:2140–50. doi: 10.3168/jds.2020-19153, PMID: 33309348

[5] Kandeel SA Megahed AA Ebeid MH Constable PD. Evaluation of 3 esterase tests for the diagnosis of subclinical mastitis at dry-off and freshening in dairy cattle. J Dairy Sci. (2019) 102:1402–16. doi: 10.3168/jds.2017-14345, PMID: 30591327

[6] Ali A Mir MUR Ganie SA Mushtaq S Bukhari SI Alshehri S. Milk-compositional study of metabolites and pathogens in the milk of bovine animals affected with subclinical mastitis. Molecules. (2022) 27:8631. doi: 10.3390/molecules27238631, PMID: 36500724 PMC9738622

[7] Burmańczuk A Hola P Wojciechowska B Kowalski C Grabowski T. Validation of relationship between milk resistance and daily yield of dairy cows. 10.1515/pjvs-2017-0092 29611639

[8] Ebrahimi M Mohammadi-Dehcheshmeh M Ebrahimie E Petrovski KR. Comprehensive analysis of machine learning models for prediction of sub-clinical mastitis: deep learning and gradient-boosted trees outperform other models. Comput Biol Med. (2019) 114:103456. doi: 10.1016/j.compbiomed.2019.103456, PMID: 31605926

[9] Bobbo T Matera R Pedota G Manunza A Cotticelli A Neglia G. Exploiting machine learning methods with monthly routine milk recording data and climatic information to predict subclinical mastitis in Italian Mediterranean buffaloes. J. Dairy Sci. (2023) 106:1942–52. doi: 10.3168/jds.2022-22292 36586801

[10] van den Borne BHP Vernooij JCM Lupindu AM van Schaik G Frankena K Lam TJGM. Relationship between somatic cell count status and subsequent clinical mastitis in Dutch dairy cows. Prev Vet Med. (2011) 102:265–73. doi: 10.1016/j.prevetmed.2011.07.013, PMID: 21885136

[11] Sumon SMMR Parvin MS Ehsan MA Islam MT. Relationship between somatic cell counts and subclinical mastitis in lactating dairy cows. Vet World. (2020) 13:1709–13. doi: 10.14202/vetworld.2020.1709-1713, PMID: 33061248 PMC7522932

[12] Ebrahimie E Mohammadi-Dehcheshmeh M Laven R Petrovski K. Rule discovery in milk content towards mastitis diagnosis: dealing with farm heterogeneity over multiple years through classification based on associations. Animals. (2021) 11:1638. doi: 10.3390/ani11061638, PMID: 34205858 PMC8227403

[13] Mammadova N Keskin İ. Application of the support vector machine to predict subclinical mastitis in dairy cattle. Sci World J. (2013) 2013:1–9. doi: 10.1155/2013/603897, PMID: 24574862 PMC3886278

[14] Baştan A Salar S Cengi̇ ZM Darbaz İ Demi̇rel MA Özen D. The prediction of the prevalence and risk factors for subclinical heifermastitis in Turkish dairy farms. Turk J Vet Anim Sci. (2015) 39:682–7. doi: 10.3906/vet-1501-80

[15] Zhou X Xu C Wang H Xu W Zhao Z Chen M. The early prediction of common disorders in dairy cows monitored by automatic systems with machine learning algorithms. Animals. (2022) 12:1251. doi: 10.3390/ani12101251, PMID: 35625096 PMC9137925

[16] Moayedi H Nguyen H Kok FL. Nonlinear evolutionary swarm intelligence of grasshopper optimization algorithm and gray wolf optimization for weight adjustment of neural network. Eng Comput. (2021) 37:1265–75. doi: 10.1007/s00366-019-00882-2

[17] Hashim FA Hussien AG. Snake optimizer: a novel meta-heuristic optimization algorithm. Knowl Based Syst. (2022) 242:108320. doi: 10.1016/j.knosys.2022.108320

[18] Liu Q Wang P Sun J Li R Li Y. Wireless Channel prediction of GRU based on experience replay and Snake optimizer. Sensors. (2023) 23:6270. doi: 10.3390/s23146270, PMID: 37514564 PMC10385616

[19] Li Y Xiao L Tang B Liang L Lou Y Guo X. A denoising method for ship-radiated noise based on optimized variational mode decomposition with snake optimization and dual-threshold criteria of correlation coefficient. Math Probl Eng. (2022) 2022:1–21. doi: 10.1155/2022/8024753, PMID: 39712885

[20] Alamir N Kamel S Megahed TF Hori M Abdelkader SM. Energy management of multi-microgrid considering demand response using snake optimizer In: 2022 23rd international Middle East power systems conference (MEPCON). Cairo, Egypt: IEEE (2022). 1–6.

[21] Pakrashi A Ryan C Guéret C Berry DP Corcoran M Keane MT. Early detection of subclinical mastitis in lactating dairy cows using cow-level features. J Dairy Sci. (2023) 106:4978–90. doi: 10.3168/jds.2022-22803, PMID: 37268591

[22] Zhou H Wang X Zhu R. Feature selection based on mutual information with correlation coefficient. Appl. Intell. (2022) 52:5457–74. doi: 10.1007/s10489-021-02524-x

[23] Rumelhart DE Hinton GE Williams RJ. Learning representations by back-propagating errors. Nature. (1986) 323:533–6. doi: 10.1038/323533a0

[24] Zhang Y Cui N Feng Y Gong D Hu X. Comparison of BP, PSO-BP and statistical models for predicting daily global solar radiation in arid Northwest China. Comput Electron Agric. (2019) 164:104905. doi: 10.1016/j.compag.2019.104905

[25] Li J Dong X Ruan S Shi L. A parallel integrated learning technique of improved particle swarm optimization and BP neural network and its application. Sci. Rep. (2022) 12:19325. doi: 10.1038/s41598-022-21463-2 36369241 PMC9652340

[26] Li Y Han M Guo Q. Modified whale optimization algorithm based on tent chaotic mapping and its application in structural optimization. KSCE J Civ Eng. (2020) 24:3703–13. doi: 10.1007/s12205-020-0504-5

[27] Hu G Yang R Abbas M Wei G. BEESO: multi-strategy boosted Snake-inspired optimizer for engineering applications. J Bionic Eng. (2023) 20:1791–827. doi: 10.1007/s42235-022-00330-w

[28] Schwarz D Diesterbeck US Failing K König S Brügemann K Zschöck M. Somatic cell counts and bacteriological status in quarter foremilk samples of cows in Hesse, Germany—a longitudinal study. J Dairy Sci. (2010) 93:5716–28. doi: 10.3168/jds.2010-3223, PMID: 21094743

