# Supplementary material for: TBESO-BP: an improved regression model for predicting subclinical mastitis
Source: Front Vet Sci. 2025 Apr 1;12:1396799. doi: 10.3389/fvets.2025.1396799 (PMC11997978; doi:10.3389/fvets.2025.1396799)
Supplement: Supplementary file 1 [file Data_Sheet_1.pdf]

Supplement Materials

1 The Process of BP Regression Prediction

For accurate regression predictions in subclinical mastitis diagnosis, a robust modeling approach is essential. This study explores the use of a Backpropagation (BP) neural network, a nonlinear regression model capable of capturing complex relationships between variables as Supplementary Figure 1.

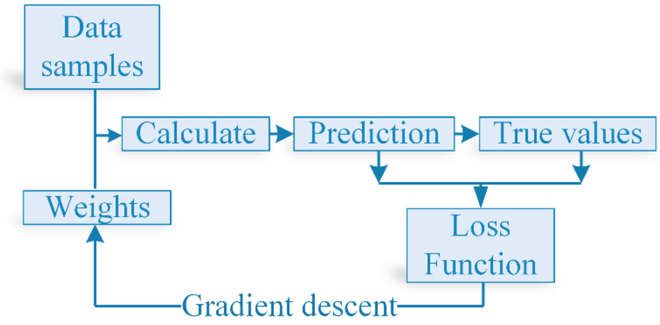

Supplementary Figure 1. The Process of BP regression prediction

The BP neural network model consists of an input layer, a hidden layer, and an output layer. For each layer in the neural network, the input is first weighted, a bias is added, and then passed through an activation function to obtain the output as Supplementary Figure 2.

When the input data X is an $m \times n$ matrix as $\boldsymbol{X} = \begin{bmatrix} x_{1,1} & \cdots & x_{1,i} & \cdots & x_{1,n} \\ \vdots & \ddots & \vdots & & \vdots \\ x_{j,1} & \cdots & x_{j,i} & \cdots & x_{j,n} \\ \vdots & & \vdots & \ddots & \vdots \\ x_{m,1} & \cdots & x_{m,i} & \cdots & x_{m,n} \end{bmatrix}$, where n is the number of data samples and m is the number of features for each sample, the network computes a predicted output $\boldsymbol{Y} = [\hat{y}_1, \hat{y}_2, \ldots, \hat{y}_i, \ldots, \hat{y}_n], i = 1,2, \ldots, n$. Choose a $\boldsymbol{x} = [x_1, x_2, \ldots, x_j, \ldots, x_m], j = 1,2, \ldots, m$, to calculate its predicted output $\hat{y}_i$.The general steps are as follows:

1.1 The Forword Propagation

1. Input Layer to Hidden Layer:

For each hidden node h_k in the hidden layer, the output is calculated by the weighted sum of the inputs and the bias b_k, followed by a nonlinear activation function f:

$$\begin{cases} \boldsymbol{z_k = \sum_{j=1}^{m} w_{jk} x_j + b_k \, , k = 1, 2, \ldots, s} \\ \boldsymbol{h_k = f(z_k)} \end{cases} \tag{1}$$

Where z_k is the total input received by hidden layer node h_k, representing the weighted sum of the input features, with the bias added, h_k is the output after applying an activation function of the k-th neuron in the hidden layer, w_{jk} is the weight between the j-th feature and the k-th hidden neuron,x_j is the j-th features for i-th sample, b_k is the bias term for the k-th hidden neuron , s represents the number of hidden neurons and $f(\cdot)$ is the non-linear activation function, this study use a hyperbolic tangent function, defined as:

$$\begin{cases} \boldsymbol{f(x) = tanh(x) = \dfrac{e^x - e^{-x}}{e^x + e^{-x}}} \\ \boldsymbol{f'(x) = tanh'(x) = \left(\dfrac{e^x - e^{-x}}{e^x + e^{-x}}\right)' = 1 - tanh^2(x)} \end{cases} \tag{2}$$

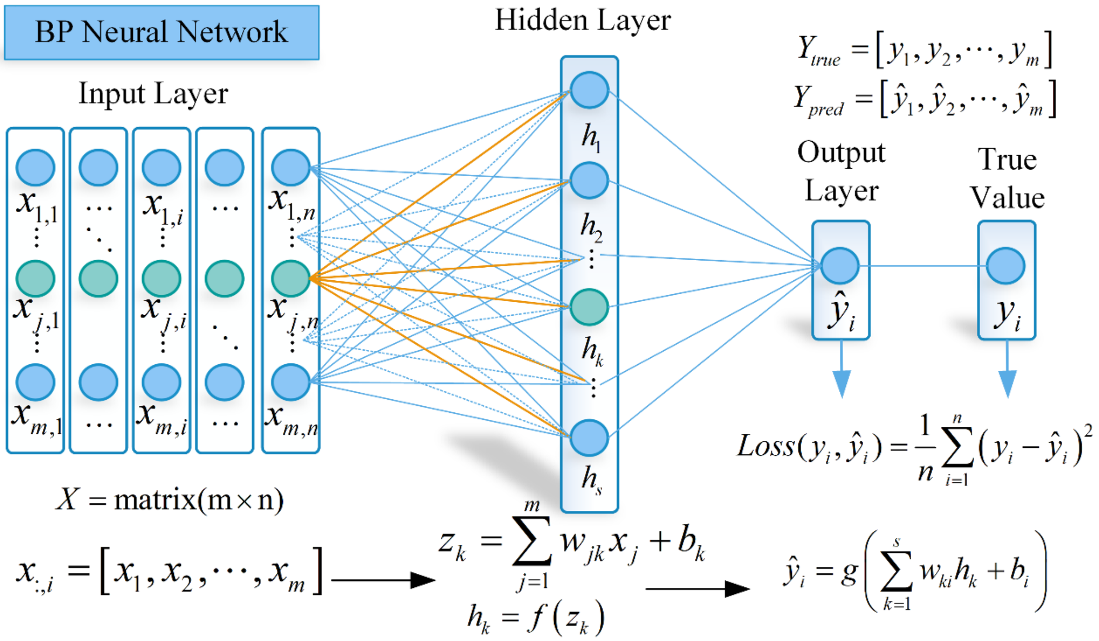

Supplementary Figure 2. The Process of BP Neural Network

2. Hidden Layer to Output Layer.

For the i-th neuron in the output layer, the output is calculated as:

$$\boldsymbol{\hat{y}_i = g\left(\sum\nolimits_{k=1}^{s} w_{ki}h_k + b_i\right)} \tag{3}$$

Where y_i is the output of the i-th neuron in the output layer, represents the predicted value of i-th sample, s is the number of neurons in the hidden layer, w_{ki} is the weight between the k-th hidden neuron and the i-th output neuron, h_k is the output of the k-th neuron in the hidden layer, b_i is the bias term for the i-th output layer neuron and $f(\cdot)$ is the non-linear activation function, this study use a liner function, defined as: $g(x) = x$.

3. The process continues layer by layer, from the input layer to the hidden layers, and finally to the output layer, where the predicted output $\hat{y}_i$ is produced, expressed as:

$$\hat{\boldsymbol{y}}_i = \boldsymbol{g}\left(\sum_{k=1}^{s} \boldsymbol{w}_{ki} \cdot \boldsymbol{f}\left(\sum_{j=1}^{m} \boldsymbol{w}_{jk}\boldsymbol{x}_j + \boldsymbol{b}_k\right) + \boldsymbol{b}_i\right), \boldsymbol{k} = \mathbf{1}, \mathbf{2}, \dots, \boldsymbol{s} \tag{4}$$

1.2 Backpropagation to Adjust Weights and Bias

Once the output $\hat{y}_i$ is computed for each data sample, the error (difference between the predicted and actual outputs) is calculated using a loss function, commonly the Mean Squared Error (MSE) for regression tasks. The goal is to minimize this error by updating the weights and biases using the backpropagation algorithm.

$$\boldsymbol{Loss}(\boldsymbol{y}_i, \hat{\boldsymbol{y}}_i) = \frac{\mathbf{1}}{\boldsymbol{n}}\sum_{i=1}^{n} (\boldsymbol{y}_i - \hat{\boldsymbol{y}}_i)^2 \tag{5}$$

where $y_i - \hat{y}_i$ represents the difference between the actual value and the predicted value on the test set for a specific data sample.

To minimize the prediction error, backpropagation is used to adjust the weights w_{ij} and biases b_i of the network based on the gradient of the error function. The gradient descent method is commonly used by updating network parameters based on the gradient information of the error function, gradually reducing the error. The update principle is as follows:

1. Output layer

For the output layer, we compute the gradient of the loss function with respect to the output neuron's weights and bias:

$$\begin{cases} \dfrac{\partial \boldsymbol{Loss}}{\partial \boldsymbol{w}'_i} = \dfrac{\mathbf{2}}{\boldsymbol{n}}\sum_{i=1}^{n} (\hat{\boldsymbol{y}}_i - \boldsymbol{y}_i)\, \boldsymbol{h}_i \\ \dfrac{\partial \boldsymbol{Loss}}{\partial \boldsymbol{b}'} = \dfrac{\mathbf{2}}{\boldsymbol{n}}\sum_{i=1}^{n} (\hat{\boldsymbol{y}}_i - \boldsymbol{y}_i) \end{cases} \tag{6}$$

2. Hidden layer

Next, we propagate the error backward through the network to the hidden layer. The gradients of the weights in the hidden layer are computed using the derivative of the loss with respect to the outputs of the hidden layer. Using the chain rule:

$$\begin{cases} \dfrac{\partial \boldsymbol{Loss}}{\partial \boldsymbol{w}_{ij}} = \dfrac{\mathbf{2}}{\boldsymbol{n}}\sum_{i=1}^{n} (\hat{\boldsymbol{y}}_i - \boldsymbol{y}_i)\, \boldsymbol{w}'_i \boldsymbol{h}_i (\mathbf{1} - \boldsymbol{h}_i^2) \boldsymbol{x}_j \\ \dfrac{\partial \boldsymbol{Loss}}{\partial \boldsymbol{b}_i} = \dfrac{\mathbf{2}}{\boldsymbol{n}}\sum_{i=1}^{n} (\hat{\boldsymbol{y}}_i - \boldsymbol{y}_i)\, \boldsymbol{w}'_i \boldsymbol{h}_i (\mathbf{1} - \boldsymbol{h}_i^2) \end{cases} \tag{7}$$

w'_i is the weight between the hidden layer and the output layer, h_i is the output of the hidden neuron, $(1 - h_i^2)$ is the derivative of the tanh activation function as Supplementary Equation (2), x_j is the input to the hidden neuron.

3. Weight Update:

After calculating the gradients, the weights and biases are updated using a gradient descent algorithm:

$$\begin{cases} \boldsymbol{w_{ij} = w_{ij} - \eta \frac{\partial Loss}{\partial w_{ij}}} \\ \boldsymbol{b_i = b_i - \eta \frac{\partial Loss}{\partial b_i}} \end{cases} \tag{8}$$

where η is the learning rate, which controls how large a step the network takes toward minimizing the error.

But gradient descent can converge slowly if the rate is too small or overshoot if it's too large. To overcome these issues, intelligent optimization algorithms, are used to determine the weights and biases. These methods don't rely on gradients and can better handle complex, nonlinear problems, offering faster convergence and more reliable results.

The gradient descent method is commonly used by updating network parameters based on the gradient information of the error function, gradually reducing the error. But gradient descent can converge slowly if the rate is too small or overshoot if it's too large. In this study, to better adapt and fit Dairy Herd Improvement (DHI) data and to improve the accuracy of the prediction model, an enhanced optimization algorithm is utilized to replace the gradient descent method of selecting the weights and bias of the BP neural network. In other words, transforming the issue of selecting the neural network's weights and thresholds into an optimization problem. That means the higher the fitness value obtained by the optimization algorithm, the smaller the corresponding loss function value, resulting in a better prediction as follows:

$$\boldsymbol{-fitness(X_i) = Loss(y_i, \hat{y}_i)} \tag{9}$$

2 Ablation Experiment

The purpose of this section is to analyze the impact of the introduced strategies on SO and their synergistic effects. TBESO combines three strategies, namely TCM, BDS, and EOBL. Each combination of these three strategies with SO results in three variants. Similarly, introducing random combinations of two strategies in SO also generates three variants. Supplementary Table 1. lists all possible variants of SO under the influence of the three strategies, where 1 and 0 indicate whether the strategy is introduced or not. For example, BE-SO in the table represents an improved SO with the addition of BDS and EOBL on top of the base SO.

Supplementary Table 1. Various SO variants with the three strategies

Strategies	**TCM-SO**	**BDS-SO**	**EOBL-SO**	**TB-SO**	**TE-SO**	**BE-SO**	**TBESO**
**TCM**	1	0	0	1	1	0	1
**BDS**	0	1	0	1	0	1	1
**EOBL**	0	0	1	0	1	1	1

All variants and SO are compared on the CEC2017 test set, which includes functions covering four types of benchmark cases: unimodal, multimodal, hybrid, and composite. Unimodal functions are used to verify the efficiency and local exploration ability of the optimization algorithm, multimodal

functions are employed to assess the algorithm's exploration capability and ability to avoid local optima, hybrid functions are designed to test the algorithm's adaptability when facing regions with different characteristics, and composite functions evaluate the overall performance of the algorithm in handling complex problems as they encompass characteristics of different types of functions. In the experiments, the dimensions for F1~F30 are all set to 30, and the results are presented in Supplementary Figure 3.

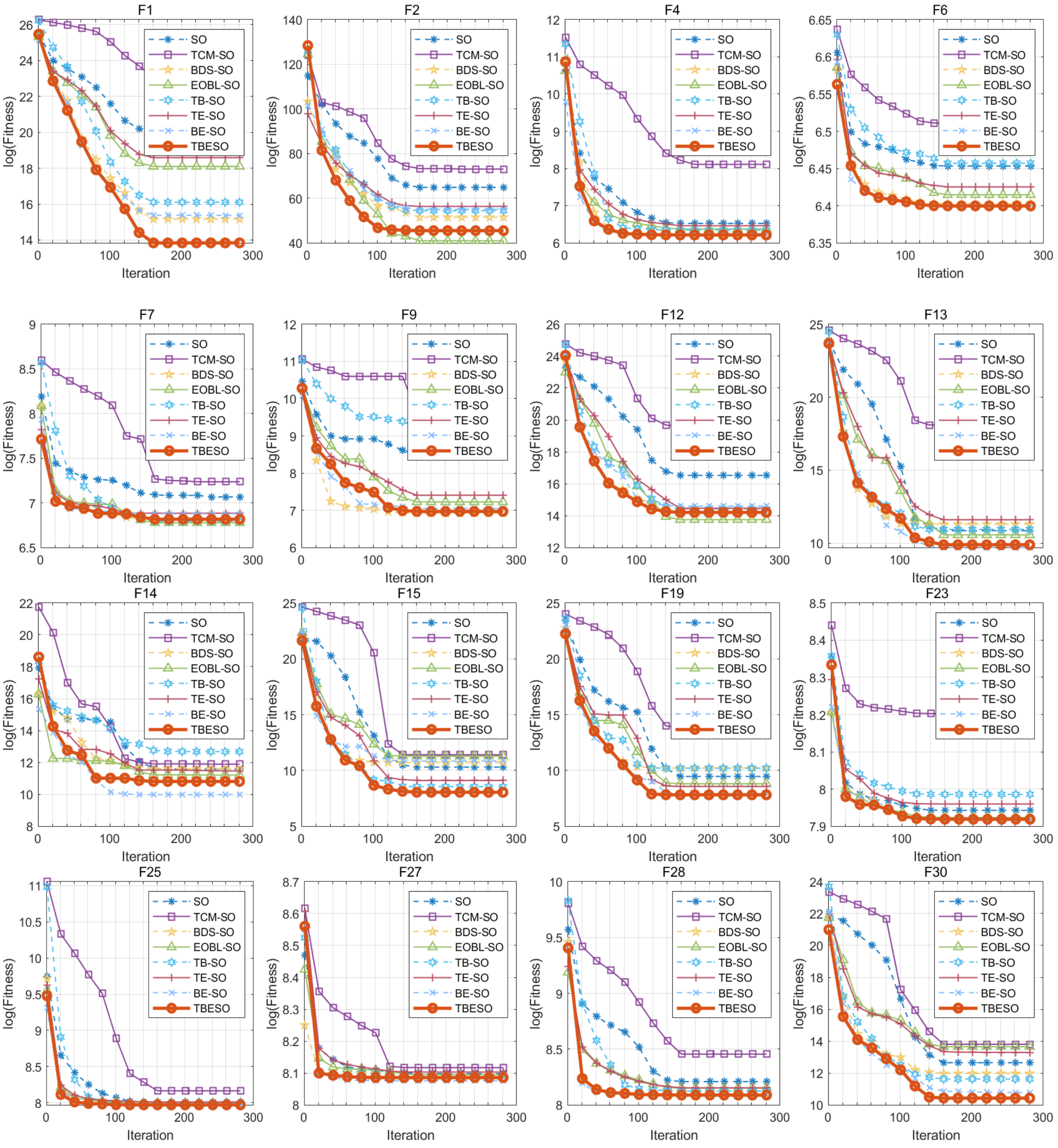

Supplementary Figure 3. Convergence curves of SO variants on some benchmark test functions

The convergence curves in Supplementary Figure 3 demonstrate that BDS-SO accelerates convergence early on, quickly guiding the algorithm toward optimal values. TBESO further enhances this with fast, stable convergence across the 16 benchmark functions, efficiently finding solutions close to the global optimum.

Compared to other SO variants, TBESO requires fewer iterations to achieve optimal results, showing strong adaptability and generalization. The data shows that all seven variants outperform the original SO, with EOBL-SO and BDS-SO standing out among single-strategy variants for their effectiveness in unimodal functions F1-F3.

The last two rows of the table summarize the average ranking, total ranking, and Friedman test ranking of the algorithms, where "−/=/+" indicates whether these variants are significantly worse than, equal to, or better than SO. Friedman test and Wilcoxon rank-sum test are used to assess the algorithm's variability, and the statistical results are shown in Supplementary Table 2.

The evaluation metrics shown in Supplementary Figure 3 include Mean, Std, and Rank, with the best Mean and Std highlighted in bold. The table summarizes the average ranking, total ranking, and Friedman test ranking of the algorithms, where "−/=/+" indicates whether these variants are significantly worse than, equal to, or better than SO.

Supplementary Table 2. Comparison results of various SO variants using CEC2017 and Dim=30.

Fun	Index	Algorithms							
		SO	TCM-SO	BDS-SO	EOBL-SO	TB-SO	TE-SO	BE-SO	TBESO
F1	Mean	2.30E+09	2.60E+10	3.57E+07	4.95E+08	4.28E+08	4.86E+08	2.60E+07	**2.44E+07**
	Std	1.03E+09	6.78E+09	2.04E+07	2.79E+08	2.07E+08	2.60E+08	**1.41E+07**	1.49E+07
F2	Mean	1.14E+32	5.58E+37	3.62E+28	6.00E+31	7.79E+30	7.61E+32	6.81E+27	**1.02E+27**
	Std	3.63E+32	2.46E+38	1.03E+29	2.32E+32	3.72E+31	4.16E+33	3.23E+28	**3.20E+27**
F3	Mean	8.13E+04	8.89E+04	7.94E+04	7.15E+04	9.15E+04	7.24E+04	6.95E+04	**6.66E+04**
	Std	8.53E+03	**6.06E+03**	1.06E+04	7.40E+03	6.96E+03	6.11E+03	1.01E+04	9.55E+03
F4	Mean	7.89E+02	3.37E+03	5.55E+02	6.48E+02	6.36E+02	6.61E+02	5.57E+02	**5.44E+02**
	Std	1.29E+02	1.15E+03	4.99E+01	6.30E+01	5.58E+01	8.16E+01	3.83E+01	**2.72E+01**
F5	Mean	6.29E+02	9.59E+02	6.69E+02	**6.06E+02**	7.81E+02	6.13E+02	6.48E+02	6.56E+02
	Std	3.03E+01	**1.59E+01**	3.94E+01	2.63E+01	4.68E+01	3.01E+01	3.86E+01	3.52E+01
F6	Mean	6.28E+02	6.67E+02	6.05E+02	6.16E+02	6.45E+02	6.15E+02	6.04E+02	**6.04E+02**
	Std	5.90E+00	6.31E+00	2.77E+00	5.09E+00	8.90E+00	4.70E+00	**1.65E+00**	2.65E+00
F7	Mean	1.10E+03	1.39E+03	9.52E+02	9.46E+02	9.73E+02	9.62E+02	9.46E+02	**9.43E+02**
	Std	5.27E+01	6.22E+01	3.75E+01	2.99E+01	6.35E+01	3.23E+01	3.04E+01	**2.89E+01**
F8	Mean	9.24E+02	1.18E+03	9.47E+02	**8.97E+02**	1.08E+03	8.97E+02	9.42E+02	9.40E+02
	Std	2.80E+01	**1.02E+01**	3.92E+01	2.15E+01	5.59E+01	2.42E+01	2.85E+01	3.58E+01
F9	Mean	4.78E+03	1.21E+04	1.67E+03	1.97E+03	1.19E+04	1.95E+03	**1.34E+03**	1.35E+03
	Std	1.43E+03	1.16E+03	5.13E+02	4.90E+02	1.61E+03	5.72E+02	4.49E+02	**3.19E+02**
F10	Mean	8.16E+03	**5.72E+03**	8.39E+03	8.59E+03	6.21E+03	8.75E+03	8.55E+03	8.56E+03
	Std	9.72E+02	9.11E+02	6.37E+02	3.65E+02	1.09E+03	**2.57E+02**	3.50E+02	4.92E+02
F11	Mean	2.14E+03	8.89E+03	1.86E+03	1.90E+03	2.09E+03	1.89E+03	1.62E+03	**1.60E+03**
	Std	4.11E+02	3.17E+03	3.24E+02	2.65E+02	4.15E+02	3.87E+02	2.09E+02	**1.90E+02**
F12	Mean	3.23E+07	4.37E+08	5.20E+06	2.00E+07	8.60E+06	1.33E+07	**4.72E+06**	5.37E+06

	Std	3.05E+07	3.66E+08	4.36E+06	1.64E+07	5.17E+06	1.23E+07	**3.32E+06**	5.86E+06
F13	Mean	4.08E+05	6.99E+07	9.50E+04	1.05E+05	1.16E+05	3.18E+05	**7.33E+04**	8.06E+04
	Std	8.38E+05	1.45E+08	1.01E+05	7.11E+04	7.30E+04	1.04E+06	**4.24E+04**	5.92E+04
F14	Mean	2.34E+05	3.67E+05	1.11E+05	2.34E+05	2.84E+05	1.78E+05	1.11E+05	**8.05E+04**
	Std	2.36E+05	4.63E+05	1.01E+05	3.05E+05	2.63E+05	2.00E+05	1.13E+05	**6.79E+04**
F15	Mean	2.90E+04	2.05E+05	2.89E+04	2.57E+04	3.07E+04	2.77E+04	**1.64E+04**	1.85E+04
	Std	2.18E+04	3.85E+05	2.08E+04	2.66E+04	1.68E+04	3.02E+04	**9.92E+03**	2.04E+04
F16	Mean	2.80E+03	4.04E+03	3.12E+03	2.97E+03	3.00E+03	**2.75E+03**	3.17E+03	3.28E+03
	Std	**3.64E+02**	4.25E+02	4.63E+02	4.59E+02	4.71E+02	3.66E+02	4.22E+02	3.89E+02
F17	Mean	2.41E+03	2.61E+03	2.22E+03	2.22E+03	2.14E+03	**2.11E+03**	2.16E+03	2.17E+03
	Std	2.81E+02	2.59E+02	2.22E+02	1.80E+02	**1.58E+02**	1.89E+02	2.16E+02	1.82E+02
F18	Mean	3.88E+06	9.25E+05	2.00E+06	1.83E+06	**4.60E+05**	1.87E+06	2.94E+06	1.28E+06
	Std	4.20E+06	1.09E+06	1.67E+06	1.76E+06	**3.20E+05**	1.69E+06	2.77E+06	1.37E+06
F19	Mean	5.53E+04	4.25E+06	2.24E+04	3.65E+04	4.59E+04	3.73E+04	1.74E+04	**1.04E+04**
	Std	9.43E+04	7.24E+06	1.93E+04	7.17E+04	1.47E+04	5.31E+04	1.89E+04	**9.88E+03**
F20	Mean	2.69E+03	3.21E+03	2.61E+03	2.65E+03	2.83E+03	**2.54E+03**	2.65E+03	2.55E+03
	Std	2.44E+02	**1.11E+02**	2.27E+02	1.62E+02	1.55E+02	2.37E+02	2.09E+02	2.47E+02
F21	Mean	2.44E+03	2.78E+03	2.46E+03	2.41E+03	2.51E+03	**2.41E+03**	2.46E+03	2.46E+03
	Std	2.51E+01	2.71E+01	4.22E+01	2.56E+01	1.80E+02	**2.21E+01**	3.91E+01	4.04E+01
F22	Mean	7.85E+03	7.07E+03	5.60E+03	2.52E+03	7.38E+03	2.79E+03	**2.40E+03**	2.76E+03
	Std	2.49E+03	6.63E+02	3.74E+03	**9.24E+01**	1.18E+03	3.17E+02	1.77E+02	1.00E+03
F23	Mean	2.86E+03	3.58E+03	2.80E+03	**2.79E+03**	2.85E+03	2.82E+03	2.80E+03	2.80E+03
	Std	5.84E+01	7.77E+01	2.84E+01	**2.74E+01**	4.08E+01	3.12E+01	4.53E+01	3.37E+01
F24	Mean	3.01E+03	3.62E+03	3.02E+03	**2.98E+03**	3.23E+03	2.99E+03	3.02E+03	3.02E+03
	Std	5.00E+01	2.05E+02	2.80E+01	4.08E+01	1.34E+02	3.10E+01	**2.59E+01**	3.27E+01
F25	Mean	3.10E+03	3.94E+03	2.95E+03	2.99E+03	3.02E+03	3.00E+03	**2.93E+03**	2.94E+03
	Std	7.59E+01	3.25E+02	3.43E+01	3.79E+01	4.54E+01	4.03E+01	2.48E+01	**1.70E+01**
F26	Mean	6.12E+03	6.33E+03	5.02E+03	5.32E+03	**3.67E+03**	5.69E+03	5.02E+03	4.62E+03
	Std	6.83E+02	1.23E+03	4.51E+02	4.08E+02	**9.60E+01**	3.35E+02	5.10E+02	9.34E+02
F27	Mean	3.31E+03	3.38E+03	3.24E+03	3.30E+03	3.24E+03	3.31E+03	**3.23E+03**	3.24E+03
	Std	4.53E+01	6.96E+01	1.73E+01	4.15E+01	**1.53E+01**	4.39E+01	1.81E+01	1.58E+01
F28	Mean	3.79E+03	5.58E+03	3.35E+03	3.48E+03	3.74E+03	3.48E+03	**3.34E+03**	3.35E+03
	Std	2.86E+02	3.63E+02	4.87E+01	7.91E+01	1.66E+02	9.87E+01	3.46E+01	**3.43E+01**
F29	Mean	4.27E+03	5.73E+03	**3.90E+03**	4.01E+03	4.31E+03	4.00E+03	3.93E+03	3.93E+03
	Std	2.96E+02	5.53E+02	2.26E+02	**1.67E+02**	2.04E+02	1.77E+02	1.72E+02	2.16E+02
F30	Mean	1.19E+06	1.10E+07	1.72E+05	8.10E+05	2.93E+05	7.13E+05	**1.42E+05**	2.04E+05
	Std	1.66E+06	1.85E+07	1.79E+05	7.63E+05	1.82E+05	5.40E+05	**1.27E+05**	1.94E+05
Friedman rank		5.30	7.27	3.87	3.83	5.40	3.90	3.60	2.83
-/=/+		/	24/1/5	5/0/25	2/2/26	9/3/18	1/3/26	4/0/26	4/1/25
Average rank		6.03	6.83	3.67	3.50	5.07	3.70	2.43	2.70
Total rank		7	8	4	3	6	5	2	1

In Supplementary Table 2, TBESO ranks first in 40% of tested functions, with an average ranking of 2.70. Wilcoxon rank-sum tests confirm TBESO's superior performance across 25 functions, achieving the highest overall Friedman ranking of 2.83. This solidifies TBESO as the best-performing variant among all tested.

3 Population Distribution

The population distribution of the TBESO is examined after 25 iterations in a two-dimensional space. The population size (N) is set to 400, with the upper boundary (UB) and lower boundary (LB) defined as 1 and -1, respectively. The distribution of the population is visualized to demonstrate the evolution of candidate solutions over the iterations as

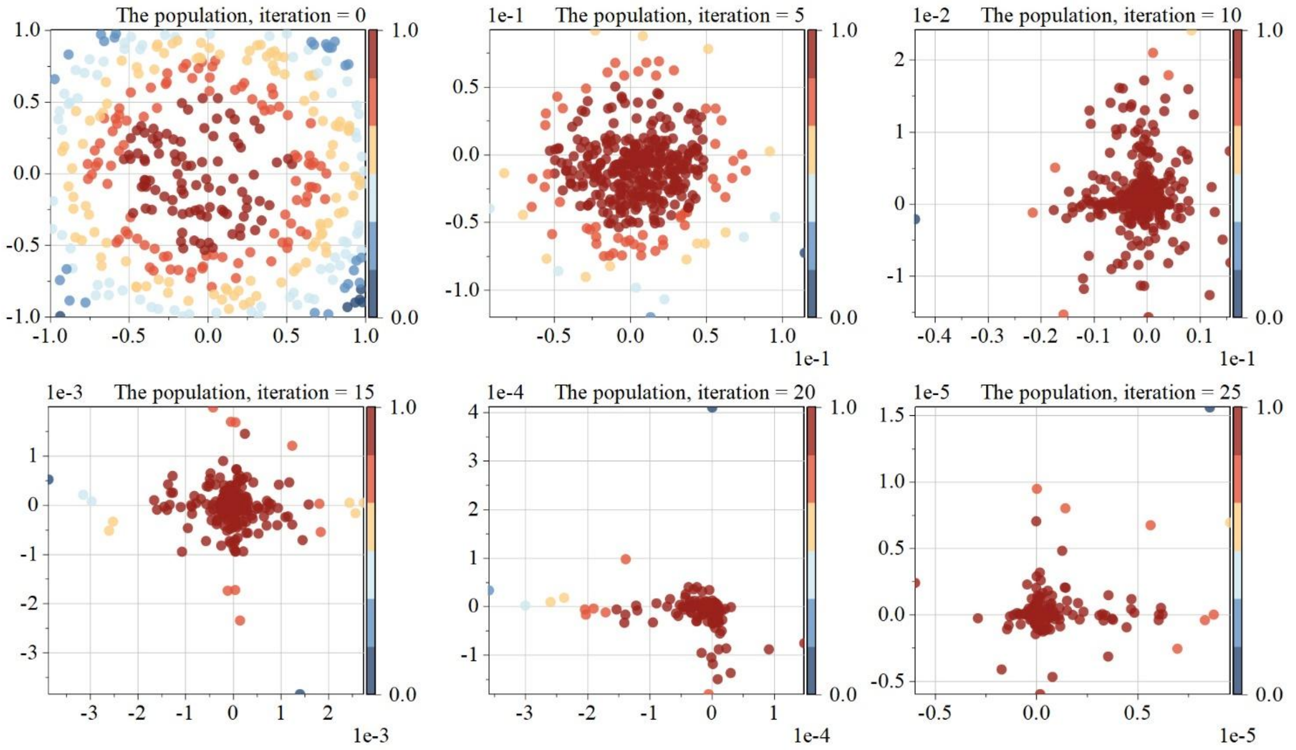

Supplementary Figure 4. Population distribution under different iteration numbers

The Ablation Experiment and the distribution charts both serve to illustrate the performance and behavior of the TBESO algorithm under different conditions. The Ablation Experiment helps in understanding the impact of various components or modifications to the algorithm by systematically removing or altering certain features. This analysis aids in evaluating the significance of each component in contributing to the overall performance.

On the other hand, the population distribution charts provide a visual representation of how candidate solutions evolve throughout the algorithm's iterations. By displaying the population's spread across the search space, these charts highlight how the algorithm converges toward the optimal solution. They show the exploration and exploitation balance achieved by the algorithm, indicating how effectively it searches for solutions within the defined boundaries. Together, these elements offer a comprehensive view of the algorithm's functionality, efficiency, and adaptability.
